# Genome-Wide Association Analysis and Genomic Prediction for Adult-Plant Resistance to Septoria Tritici Blotch and Powdery Mildew in Winter Wheat

**DOI:** 10.3389/fgene.2021.661742

**Published:** 2021-05-12

**Authors:** Admas Alemu, Gintaras Brazauskas, David S. Gaikpa, Tina Henriksson, Bulat Islamov, Lise Nistrup Jørgensen, Mati Koppel, Reine Koppel, Žilvinas Liatukas, Jan T. Svensson, Aakash Chawade

**Affiliations:** ^1^Department of Plant Breeding, Swedish University of Agricultural Sciences, Alnarp, Sweden; ^2^Institute of Agriculture, Lithuanian Research Centre for Agriculture and Forestry (LAMMC), Kėdainiai, Lithuania; ^3^Lantmännen Lantbruk, Svalöv, Sweden; ^4^Estonian Crop Research Institute, Jõgeva, Estonia; ^5^Department of Agroecology, Aarhus University, Slagelse, Denmark; ^6^Estonian University of Life Sciences, Tartu, Estonia; ^7^Nordic Genetic Resource Center, Alnarp, Sweden

**Keywords:** wheat, Septoria tritici blotch, powdery mildew, GWAS, genomic prediction, genebank

## Abstract

Septoria tritici blotch (STB) caused by the fungal pathogen *Zymoseptoria tritici* and powdery mildew (PM) caused by *Blumeria graminis* f.sp *tritici* (*Bgt*) are among the forefront foliar diseases of wheat that lead to a significant loss of grain yield and quality. Resistance breeding aimed at developing varieties with inherent resistance to STB and PM diseases has been the most sustainable and environment-friendly approach. In this study, 175 winter wheat landraces and historical cultivars originated from the Nordic region were evaluated for adult-plant resistance (APR) to STB and PM in Denmark, Estonia, Lithuania, and Sweden. Genome-wide association study (GWAS) and genomic prediction (GP) were performed based on the adult-plant response to STB and PM in field conditions using 7,401 single-nucleotide polymorphism (SNP) markers generated by 20K SNP chip. Genotype-by-environment interaction was significant for both disease scores. GWAS detected stable and environment-specific quantitative trait locis (QTLs) on chromosomes 1A, 1B, 1D, 2B, 3B, 4A, 5A, 6A, and 6B for STB and 2A, 2D, 3A, 4B, 5A, 6B, 7A, and 7B for PM adult-plant disease resistance. GP accuracy was improved when assisted with QTL from GWAS as a fixed effect. The GWAS-assisted GP accuracy ranged within 0.53–0.75 and 0.36–0.83 for STB and PM, respectively, across the tested environments. This study highlights that landraces and historical cultivars are a valuable source of APR to STB and PM. Such germplasm could be used to identify and introgress novel resistance genes to modern breeding lines.

## Introduction

Septoria tritici blotch (STB) and powdery mildew (PM) caused by fungal pathogens *Zymoseptoria tritici* and *Blumeria graminis* f.sp *tritici* (*Bgt*), respectively, are the major devastating foliar diseases that cause significant yield loss in wheat-growing regions. These fungal diseases can significantly reduce the yield and quality of the wheat crop under conducive environmental conditions (Singh et al., 2016; Figueroa et al., 2018; Jalli et al., 2020). Both STB and PM diseases are major concerns for sustainable wheat production in European countries around the Baltic Sea (Chawade et al., 2018). In this region, fungicides are widely used for the control of these diseases. However, the growth of pathogen strains with resistance/insensitivity to the existing widely used fungicides (Heick et al., 2017) coupled with the worrisome environmental impact of fungicide application critically demands resistance breeding aimed at developing varieties with inherent resistance to STB and PM diseases. Increasing temperatures due to global warming and sufficient precipitation for rainfed agriculture in northern Europe are likely to increase crop productivity (Porter and Semenov, 2005; Castellari and Kurnik, 2017). Nevertheless, the milder winters and longer growing periods may encourage the spread of diseases (Wiik and Ewaldz, 2009; Roos et al., 2011). Hence, decreasing the negative effect of climate change by reducing the spread of plant diseases is immense, and in this case, landraces are the ultimate genetic resources for disease resistance breeding (Olesen et al., 2011).

Major genes with strong effect and genes with minor-to-moderate effect inherited qualitatively and quantitatively, respectively, have been reported in wheat resistance against both STB and PM. Twenty-one qualitatively inherited major genes (*Stb* genes) are detected hitherto and mapped on 14 wheat chromosomes against the STB pathogen (Brown et al., 2015). These genes are generally effective but are genotype specific, and their potency is only for a particular isolate of the pathogen (Brown et al., 2015). Their act of resistance is supposed to be a gene-for-gene relationship as demonstrated in the *Stb6* gene (Brading et al., 2002). Both *Stb6* and *Stb16q* were the source of resistance in European cultivars (Ghaffary et al., 2012) but lost their effectiveness lately in certain geographical regions (Saintenac et al., 2018; Kildea et al., 2020). These pathogen-specific major genes can confer resistance to the pathogen either at the seedling stage (*Stb7*, *Stb9-15*, *StbSm3*, *StbWW*, and *TmStb1*), adult stage (*Stb2*, *Stb3*, *Stb8*, and *Stb17*), or at all growth stages (*Stb1*, *Stb4-6*, *Stb16q*, and *Stb18*) of the wheat plant (Raman and Milgate, 2012; Brown et al., 2015). The majority of European wheat varieties encompass qualitative and quantitative genes, although several of the major genes are no more effective in fields (Arraiano and Brown, 2006). Hence, detecting and accumulating quantitative trait loci (QTLs) with minor-to-moderate effects have been evolved as an effective way to have durably resistant wheat varieties for STB. Likewise, to STB, both qualitative and quantitative genes are detected in wheat for PM resistance. So far, more than 100 *pm* alleles in 60 loci (*Pm1–Pm68: Pm8* is allelic to *Pm17*; *Pm18 = Pm1c*; *Pm22* = *Pm1e*; *Pm23* = *Pm4c*; and *Pm31* = *Pm21*) are identified and mapped across wheat chromosomes (Li et al., 2019a; Jia et al., 2020; McIntosh et al., 2020). Several of these genes are detected in Scandinavian wheat cultivars in the past, either singly or in combinations of multiple genes (Hovmøller, 1989) in addition to genes with minor effects (Keller et al., 1999; Chen et al., 2009; Lillemo et al., 2012). Such minor-effect genes are mostly quantitatively inherited and often provide sufficient levels of disease resistance for longer time compared to race-specific resistance (Limpert et al., 1987). Only three PM resistance genes (i.e., *Pm38*, *Pm39*, and *Pm46*) are identified, mapped and provide quantitative PM resistance (Spielmeyer et al., 2005; Lillemo et al., 2008; Herrera-Foessel et al., 2014). The gene *Pm3* comprised the highest numbers of alleles with 17, followed by *Pm1* and *Pm5* each contains 5, *Pm4* with 4, and *Pm2* with 3 alleles (Hao et al., 2015). The majority of these *Pm* genes were transferred from the domesticated and wild relatives of wheat and even from other species, such as rye (*Secale cereale*) (Marone et al., 2013). However, this phenomenon imposes the difficulty not to widely utilize these genes in wheat breeding because of linkage drag (Jia et al., 2020). For instance, the *Pm8* gene that originated from the 1RS chromosome of *S. cereale* significantly contributed to PM resistance of wheat in the 1990s, but the linked secalin glycopeptide in 1RS triggered a decline in flour quality (Lee et al., 1995). Besides, most of these resistance genes have been conquered by the pathogen, and only a few are still effective against the *Bgt* isolates on the field (Zeng et al., 2014). On the other hand, the quantitative resistance genes to PM, commonly observed in adult plants on the field, comprise polygenes (QTLs) with durable and broad-spectrum characteristics, making it a more suitable approach in resistance breeding programs (Kou and Wang, 2010).

With the continuous decline of genotyping cost coupled with the increasing availability of various single-nucleotide polymorphism (SNP) fingerprinting platforms, genomic-assisted breeding has become a suitable method in various plant-breeding schemes. Linkage mapping and genome-wide association study (GWAS) are successfully applied to detect QTLs/genes in various crops. The GWAS method overcomes the two common limitations (i.e., restricted allelic diversity and limited genomic resolution) raised by the bi-parental linkage mapping approach (Brachi et al., 2011). GWAS was successfully used as a tool to mine several putative QTLs/genes associated with traits of agronomic importance in several plants, including disease resistance (Bartoli and Roux, 2017; Vagndorf et al., 2017; Juliana et al., 2018; Muqaddasi et al., 2019). Several valuable QTLs/genes have been mined through either or both GWAS and linkage mapping for STB (Schilly et al., 2011; Ghaffary et al., 2012; Miedaner et al., 2013; Dreisigacker et al., 2015; Mirdita et al., 2015; Vagndorf et al., 2017; Muqaddasi et al., 2019; Odilbekov et al., 2019a) and PM (Hartl et al., 1995; Keller et al., 1999; Hysing et al., 2007; Hua et al., 2009; Hao et al., 2015; Li et al., 2019a,b; Jia et al., 2020; Nordestgaard et al., 2020) and incorporated in wheat resistance breeding programs. Genomic prediction (GP) is the other practical approach in several crop plants to predict the genotypic estimated breeding values (GEBVs) of individual genotypes based on their overall genome-wide markers effect without the need to phenotype them (Meuwissen et al., 2001; Lorenz et al., 2011; Crossa et al., 2017). Hence, unlike GWAS and linkage mapping, QTLs with minor effects could be counted in genomic selection. Regardless of the hindering challenges in accuracy, including quality of both genotyping and phenotyping, genotype-by-environment (G × E) interaction and other non-additive effects (Voss-Fels et al., 2019), GP has proven its potential to increase the genetic gain within a significantly reduced time in several plant breeding programs (Crossa et al., 2017). GP was used previously to predict the STB and PM resistance in wheat (Mirdita et al., 2015; Juliana et al., 2017; Muqaddasi et al., 2019; Odilbekov et al., 2019a; Sarinelli et al., 2019).

Wheat landraces have been used as an invaluable source of novel genes for various traits of agronomic importance. The Nordic Genetic Resource Centre (NordGen, Alnarp, Sweden) preserved winter wheat accessions comprising landraces and historical cultivars collected from the Scandinavian countries^1^. This preserved germplasm has encompassed a rich genetic variation for various traits of agronomic importance (Chawade et al., 2018), including resistance to STB at the seedling stage (Odilbekov et al., 2019a), adult-plant resistance (APR) to rust and PM (Hysing et al., 2006, 2007), and drought tolerance (Kumar et al., 2020). The current study used 175 genotypes comprising both landraces and historical cultivars of the Nordic origin with the following objectives: (I) to evaluate the genetic variation in response to STB and PM resistance; (II) to estimate the genotype-by-environment (G × E) interaction for STB and PM resistance across years and locations; (III) to detect valuable QTLs for STB and PM resistance via GWAS analysis; and (IV) to estimate the GEBVs of individual genotypes for STB and PM APR using different GP models. For this purpose, genotypes were tested for their resistance response to STB and PM in field trials in Denmark, Estonia, Lithuania, and Sweden.

## Materials and Methods

### Plant Materials

This study used 175 winter wheat genotypes comprising both cultivars and landraces. The details of genotypes can be found in Odilbekov et al. (2019a). Briefly, the panel comprised cultivars released in the years from 1900 to 2012, representing a century of winter wheat breeding in the region. The collection also included four genotypes originally from Germany but widely grown in the Scandinavian countries. The seeds were procured from the Nordic Genetic Resources Center, Alnarp, Sweden.

### Field Experiment

Field evaluation of genotypes was carried out in four different countries, including Denmark, Estonia, Lithuania, and Sweden for two growing seasons (2018–2019 and 2019–2020). Genotypes were screened under natural infections for STB and PM and scored on a 1–9 scale, which was used previously by Laidig et al. (2021). Experiments were carried out in a lattice design with two replications having a slight variation in plot and row sizes across locations. The Denmark field trial was carried out at Tystofte in 2019 with a 1-m^2^ plot size comprising six rows, and the distance between adjacent plots and rows within plots was 50 and 20 cm, respectively. Disease assessment for both STB and PM was done once at the flag leaf stage [Zadok’s growth stage (GS) 73–75] on June 26, 2019. The Estonia field trial was done at Jõgeva for two growing seasons (2018–2019 and 2019–2020). The plot size was 1 m^2^ with six rows, and the distance between adjacent plots and rows within plots was 50 and 20 cm, respectively. Both disease assessments were done twice each year (June 6 and 20 in 2019 and July 2 and 14 in 2020) on the flag leaves. The Lithuania field trial was carried out at Dotnuva for two growing seasons (2018–2019 and 2019–2020) with a plot size of 1 m^2^ encompassing six rows, and the distance between plots and rows within plots was 80 and 20 cm, respectively. The disease evaluation for PM was scored for 2 years on June 3, 2019 and June 23, 2020. However, the STB assessment was done only in 2020 for two rounds (June 23 and July 4). The field trial in Sweden was carried out in Svalöv in 2019 with a plot size of 1 m^2^ having six rows. Possible covariates such as plant height and phenology (i.e., days from planting to heading) were also recorded at all locations except Denmark.

### Phenotypic Data Analysis

The phenotypic data analysis of individual experiments (location–year combination, environment hereafter) and combined environment was evaluated using the Multi-environment Trial Analysis with R (META-R) software package (Alvarado et al., 2020). The analysis of variance (ANOVA) and adjusted means [best linear unbiased estimations (BLUEs)] of the disease traits were calculated including locations, years, replications, blocks, and genotypes. For each environment, variances and adjusted means (BLUEs) without covariates were estimated using the linear model:

$$Y_{\mathrm{ijk}}=\mu +{\mathrm{Rep}}_{i}+{\mathrm{Block}}_{j}\,\left({\mathrm{Rep}}_{i}\right)+{\mathrm{Gen}}_{k}+\varepsilon_{\mathrm{ijk}}$$

while with covariates, phenotypic data adjustment was made with the model:

$$Y_{\mathrm{ijk}}=\mu +{\mathrm{Rep}}_{i}+{\mathrm{Block}}_{j}\,\left({\mathrm{Rep}}_{i}\right)+{\mathrm{Gen}}_{k}+\mathrm{Cov}+\varepsilon_{\mathrm{ijk}}$$

where *Y****_*ijk*_*** is the disease traits, μ is the overall mean effect, and Rep***_*i*_*** and Block***_*j*_***(Rep***_*i*_***) denote the effect of the ***i***th replicate and the ***j***th incomplete block within the ***i***th replicate, respectively. Gen***_*k*_*** represents the effect of the ***k***th genotype; ε_*ijk*_ is the effect of the error associated with the **i**th replication, ***j***th incomplete block, and ***k***th genotype, which is assumed to be the independent and identically distributed random variables (iid) normal with mean zero and variance σ^2^_ε_. Cov is the effect of the covariate (plant height and/or days to heading). All, except the overall mean and covariate, are treated as random effects and the iid normal with mean zero and effect-specific variances. Genotypes were considered as fixed effects during the BLUEs estimation. Days to heading and plant height were incorporated into the analysis whenever they were significantly correlated with the disease scores. BLUEs and variance components for combined environment were estimated using the model:

$$Y_{\mathrm{ijk}}=\mu +E\,n\,v_{i}+R\,e\,p_{j}\,\left(E\,n\,v_{i}\right)+B\,l\,o\,c\,k_{k}\,\left(E\,n\,v_{i}\,R\,e\,p_{j}\right)+G\,e\,n_{l}+E\,n\,v_{i}\times G\,e\,n_{l}+\varepsilon_{i\,j\,k}$$

where Env_*i*_ and Env_*i*_ × Gen_*l*_ are the ***i***th environment and the G × E interaction effects, respectively. Environment refers to the location–year combination.

The broad-sense heritability of the combined environment analysis was calculated with the formula:

$$H^{2}=\frac{\sigma^{2}\,\text{g}}{\sigma^{2}\,\text{g}+\sigma^{2}\,\text{ge}/n\,E\,n\,v+\sigma^{2}\,e/\left(n\,E\,n\,v\,X\,n\,R\,e\,p\right)}$$

while for individual environments, repeatability among replications was calculated with the formula:

$$H=\frac{\sigma^{2}\,\text{g}}{\sigma^{2}\,\text{g}+\sigma^{2}\,\text{e}/n\,R\,e\,p}$$

where σ^2^_*g*_ and σ^2^_*e*_ are the genotype and error variance components, respectively, σ^2^_*ge*_ is the G × E interaction variance component, *n*Rep is the number of replicates, and *n*Env is the number of environments. Frequency distribution and box plots of the phenotypic data were analyzed using Minitab 18 (Minitab Ltd., Coventry, United Kingdom), while Pearson’s correlation between phenotypic traits was computed using *cor* function in R environment (R Core Team, 2020).

The genotype plus genotype-by-environment interaction (GGE) biplot analysis was conducted based on the BLUEs values of 175 Nordic winter wheat genotypes collected from four countries using the “GGEBiplots” package in the R environment (Yan and Kang, 2002).

### Genotyping, Linkage Disequilibrium, and Genome-Wide Association Analysis

Genotypic data that was previously published (Odilbekov et al., 2019a) was used in this study. A total of 7,401 quality SNP markers were used after excluding those markers with > 20% missing values and < 5% minor allele frequency per individual genotypes.

GWAS was performed with the six different models comprising single- and multi-locus analysis methods in GAPIT v.3 software package (Lipka et al., 2012). Population structure (Q) was modeled using principal component analysis (PCA) according to Price et al. (2006), and the optimal numbers of PCA were estimated based on the Bayesian information criterion (BIC) (Schwarz, 1978). The single-locus GWAS models including general linear model (GLM) with PCA (Price et al., 2006), mixed linear model (MLM) with PCA and Kinship similarity matrix (K) (Yu et al., 2006), and Settlement of MLM Under Progressively Exclusive Relationship (SUPER) (Wang et al., 2014a) were employed to execute valuable quantitative trait nucleotides (QTNs). The multi-locus GWAS models including multiple-locus mixed linear model (MLMM) (Segura et al., 2012), fixed and random model circulating probability unification (FarmCPU) (Liu et al., 2016), and Bayesian-information and linkage-disequilibrium iteratively nested keyway (BLINK) (Huang et al., 2019) were also utilized for marker–trait association (MTA) analysis. GWAS was performed using the datasets generated from the BLUEs of STB and PM disease scores based on the following scenarios: (I) individual environments, (II) country 2 years combined, (III) GGE biplot environments grouping based results, and (IV) overall combined. The *P* < 0.001 [–log_10_(*P*) > 3.0] was used as a threshold to report significant MTAs as described earlier (Maccaferri et al., 2016; Liu et al., 2017; Alemu et al., 2021).

Genome-wide linkage disequilibrium (LD) analysis was performed using the entire genotypic dataset. Pairwise squared allele-frequency correlations (*r*^2^) between SNP markers was calculated in Trait Analysis by Association, Evolution, and Linkage (TASSEL) with 100 sliding window size. To estimate the extent of LD between pairs of loci, *r*^2^ values were plotted against physical distance (cM). The LD decay curve line was then fitted on the scatterplot using the smoothing spline regression line at the genome level following the procedure by Hill and Weir (1988) in R environment with the script previously used by Marroni et al. (2011).

### Genomic Prediction

Genomic prediction was explored for each environment and environments combined using the ridge regression BLUP (RR-BLUP) mixed model package “rrBLUP” (Endelman, 2011; Endelman and Jannink, 2012) in R environment with the formula:

y=X⁢β+Z⁢μ+ε

where *X* and *Z* are the designed matrices for fixed and random effects, respectively. β and μ represent the vectors of fixed and random effects, respectively, while *y* is a vector of phenotypic values (BLUEs), and ε is the residual error. GP was further tested using a model called the weighted ridge regression BLUP (wRR-BLUP) (Zhao et al., 2014; Spindel et al., 2016). To do this, the topmost five highly significant SNP markers that are detected repeatedly in at least two models during the GWAS analysis in each/across environments was fitted as a fixed effect in the model. Hence, the *X*β factor was applied only when significant SNPs from the GWAS analysis result were fitted as fixed effects on the model.

Cross-validation was implemented to evaluate the prediction accuracy of the model. For this purpose, the dataset was divided randomly as a training and testing set and conducted for 500 iterations. The percentage of training and testing set was 80 and 20%, respectively. Predictive ability was estimated as the correlation coefficient between the observed BLUEs of the genotypes and predicted values of the test set based on the effect estimates of genotypes in the training set. Prediction accuracy was then calculated from prediction ability divided by the square root of traits’ broad sense heritability (Legarra et al., 2008; Chen et al., 2011).

## Results

### Phenotypic Analysis

**FIGURE 1:**
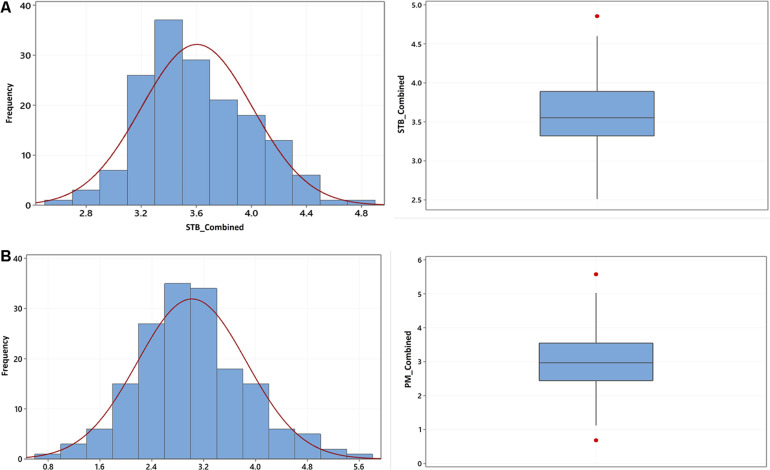
Frequency distribution **(left)** and box plot **(right)** of the BLUEs of **(A)** STB and **(B)** PM diseases scores combined from five environments using 175 winter wheat genotypes.

The phenotypic variation in STB and PM disease scores based on the individual environment and environments combined BLUEs is summarized in Table 1. Briefly, disease scores of both STB and PM showed a significant variation among genotypes for the combined as well as environment-specific analysis. The environment-combined broad sense heritability was generally higher for PM (0.69) than STB (0.39). The highest environment-specific repeatability was recorded from the Lithuania trial tested in 2020 for both STB and PM with 0.98 and 0.99 scores, respectively, while the lowest for STB was from the Denmark trial (0.39) and for PM from the Sweden trial (0.45). A normal frequency distribution was observed for both STB (Figure 1A) and PM (Figure 1B) for environment-combined BLUEs. Nonetheless, both normal and skewed types of frequency distributions were recorded on the environment-specific STB (Supplementary Figure 1) as well as PM (Supplementary Figure 2) disease scores. The genotype main effect plus genotype-by-environment interaction (GGE) biplot analysis was conducted using the scatter plot method to estimate genotype performance across tested environments. The first two principal components (PC1 and PC2) explained 67.4 and 84.1% of the total phenotypic variance for STB and PM, respectively (Supplementary Figure 3). Most of the tested genotypes had an average STB score for the tested environments and gathered together at the coordinates’ center (Supplementary Figure 3A). However, some genotypes such as Borstvete från Gotland, Ankar, and Hereford on Sweden while Äring on Denmark trial had better performance for STB resistance. Genotypes had a better departure for the PM resistance response and showed some environment preferences (Supplementary Figure 3B). Genotypes including Renodlat sammetsvete, Väinö, and Junker were more effective for PM resistance in the Lithuania trial conducted in 2020. The Estonia and Lithuania trials were positively correlated for environmental effect in both STB and PM diseases scores. The year effect was higher than the location effect on genotypes for PM scores at the Lithuania and Estonia trials (Supplementary Figure 3B).

**TABLE 1:** Analysis of variance and heritability for the two disease scores across and combined environments.

Individual environments								

Traits		EE1	EE2	LT1	LT2	SE1	DK1	
STB	Max	4.8	6.3	–	5.5	6.2	6.5	
	Min	2	2.3	–	2.5	2.5	1.5	
	Mean	3.1	3.7	–	3.8	4.1	3.0	
	σ^2^G	0.00712***	0.0248***	–	0.0312***	0.0543***	0.0254***	
	σ^2^E	0.0205	0.0131	–	0.0015	0.1368	0.0786	
	H	0.41	0.80	–	0.98	0.44	0.39	
PM	Max	7.8	–	6	6	3.5	4	
	Min	1.2	–	1	1	0.5	1	
	Mean	5.5	–	3.1	1.8	1.1	1.2	
	σ^2^G	1.5798***	–	1.0453***	1.4713***	0.0144***	0.1249***	
	σ^2^E	0.2694	–	0.0678	0.0256	0.0353	0.2260	
	H	0.92	–	0.97	0.99	0.45	0.53	

**Combined**								

**Traits**	**Mean**	**Max**	**Min**	**σ^2^G**	**σ^2^GxE**	**σ^2^*E***	**CV**	***H*${}^{2}$**

STB	2.7	5.5	3.6	0.0635**	0.2017***	0.3756	16.8	0.39
PM	2.9	5.7	0.5	0.4432***	0.7354***	0.1452	13.2	0.69

Traits: STB, septoria tritici blotch; PM, powdery mildew. Environments (location × year combined): EE, Estonia; LT, Lithuania; SE, Sweden; DK, Denmark; Years: 1, 2018/19; 2, 2019/20. Variance: σ^2^G, genetic variance; σ^2^G × E, genotype-by-environment interaction variance; σ^2^E, environmental variance; H^2^, broad sense heritability; H, repeatability.

**P < 0.01; *** *P* < 0.001.

### GWAS and LD Analysis

The GWAS analysis was conducted using the BLUEs from the two diseases using six different models including GLM, MLM, SUPER, MLMM, FarmCPU, and BLINK, in which the first three are single-locus while the last three multi-locus models. Population structure was modeled using PCA, and the optimal numbers of PCs were estimated based on the BIC. Ten PCs were initially included to select the best numbers of PCs with the highest BIC values, and the result indicated that three PCs were the optimal number to be included in the GWAS analysis models. The six models extensively detected stable and environment-specific QTNs for both STB and PM resistance across chromosomes. The detected QTNs with −log_10_(*P*) > 3.0 for each environment and combined analysis are reported in Supplementary Tables 1–8 for STB and in Supplementary Tables 9–18 for the PM, while Manhattan and Q–Q plots of the GWAS results are reported in Supplementary Figures 4A–F, 5A–F for STB and PM traits, respectively.

**FIGURE 2:**
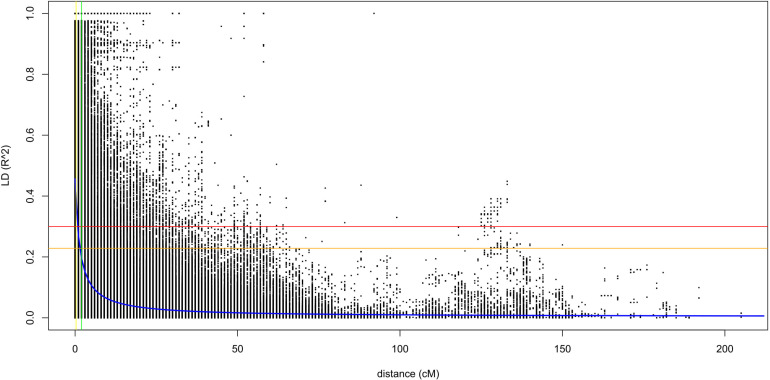
A scatter plot for *r*^2^ values of pairwise SNPs showing genome-wide linkage disequilibrium (LD) decay. The blue curve line is the smoothing spline regression model fitted to LD decay. The horizontal red line is the standard critical *r*^2^ value of the genome (*r*^2^ = 0.3), and the vertical yellow line is the genetic distance (1.6 cM) at the intersect between the standard critical and the LD decay curve. The vertical green line is the genetic distance (2.0 cM) at which the LD half-decay (*r*^2^ = 0.23, the vertical orange line) intersect with the LD decay curve.

The linkage disequilibrium analysis was done with the data generated from TASSEL in 100 sliding window size. The average *r*^2^ value of the genome was 0.12, and the LD decay started at *r*^2^ of 0.48 and reached half-decay at 0.23. The LD decay curve intersected with the half-decay and the standard critical (*r*^2^ = 0.3) at 2.0 and 1.6 cM, respectively (Figure 2). This defines the ± 1.6 cM as the genome-wide critical distance to detect linkage, and QTNs within this distance could be considered as a single QTL.

### GWAS for STB

**FIGURE 3:**
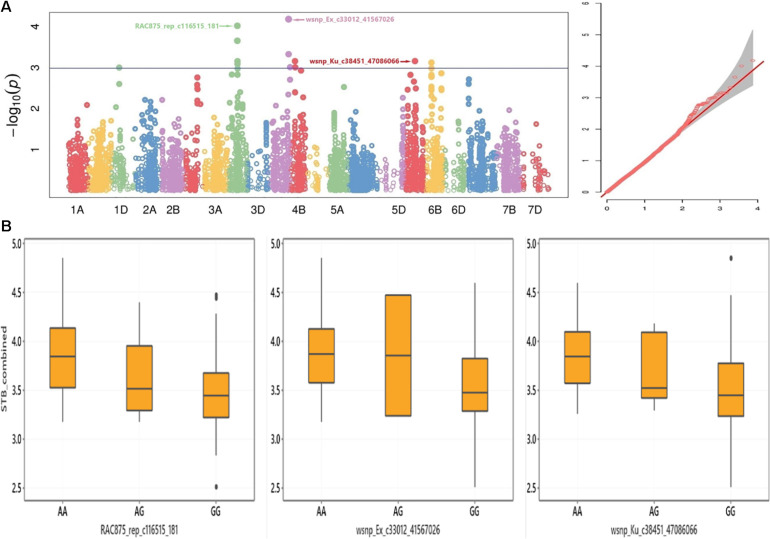
**(A)** Manhattan (left) and Q–Q (right) plots for GWAS result of adult-plant resistance response to STB and **(B)** box plots for STB resistance values grouped by alleles of the SNP markers *RAC875_rep_c116515_181*, *wsnp_Ex_c33012_41567026*, and *wsnp_Ku_c38451_47086066*.

This study discovered SNP markers with significant association across environments for the STB disease trait (*P* < 0.001). For instance, the SNP marker *wsnp_Ex_c33012_41567026* on chromosome 4A was significantly associated with STB resistance in Estonia trial 2019, Estonia 2 years combined, Estonia and Lithuania combined, as well as in all environments combined (Table 2). The alleles of this marker had particularly shown a noticeable effect on the distribution of STB combined from all the five tested environments (Figures 3A,B). SNP marker *RAC875_rep_c116515_181* on chromosome 3B had a significant association with significant allelic effect with STB mean values combined from all tested environments (Figure 3B). Individual genotypes carrying the favorable allele (i.e., guanine) on SNP markers *wsnp_Ex_c33012_41567026* and *wsnp_Ku_c38451_47086066* on chromosomes 4A and 6A decreased STB susceptibility by 4.3 and 4.4%, respectively (Figure 3B). Markers *BS00021714_51* and *RAC875_c68120_285* on chromosomes 1A and 5A, respectively, had a significant association with STB mean values scored from Estonia (2020), Estonia 2 years combined, and Estonia and Lithuania combined mean values. In addition to the marker *RAC875_c68120_285*, five markers (*BS00065313_51*, *RAC875_c31670_389*, *BobWhite_c11512_157*, *Kukri_c63163_141*, and *Excalibur_c2598_2052*) positioned within 1-cM interval had shown a significant association with STB scored from Estonia in 2020 (Supplementary Table 2 and Supplementary Figure 4A). The *wsnp_Ku_c38451_47086066* marker on chromosome 6A had a significant MTA with the STB disease scored in Sweden and all environments combined (Table 2). Similar regions on chromosome 2B were detected with SNP markers having a significant association for BLUEs STB values scored from Sweden and Denmark trials (Supplementary Tables 4,5). Chromosomes including 1B, 2B, 3B, 5A, and 5B were the sources of environment-specific QTNs for STB.

**TABLE 2:** Summary of GWAS results for significant SNP markers for Septoria tritici blotch.

Marker	Chr	Pos	Env	Models
wsnp_Ex_c33012_41567026	4A	153	E1	BLINK*, FarmCPU*, GLM*, MLM*, MLMM*, SUPER*
			E6	BLINK*, FarmCPU*, MLMM*
			E7	BLINK*, FarmCPU*, GLM*, MLM*, MLMM*, SUPER*
			E8	MLM**, SUPER**, MLMM**, GLM**, FarmCPU*, BLINK*
BS00021714_51	1A	78	E2	BLINK**, FarmCPU**, MLMM*, SUPER*
			E6	BLINK**, FarmCPU**, MLMM*, SUPER*
			E7	BLINK*, FarmCPU*
RAC875_c68120_285	5A	63	E2	MLMM**, SUPER**, BLINK*, FarmCPU*, MLM*
			E6	BLINK**, SUPER**, FarmCPU**, MLMM**, GLM*, MLM*
			E7	MLMM*, SUPER*
wsnp_Ku_c38451_47086066	6A	79	E4	SUPER**, MLMM**, GLM**, MLM*, BLINK*, FarmCPU*
			E8	BLINK*, FarmCPU*, MLMM*, GLM*, MLM*, SUPER*
wsnp_CAP11_c59_99769	3B	115	E2	GLM**
			E6	BLINK**, FarmCPU**, MLMM**, SUPER**, MLM*, GLM*
D_F5XZDLF01A85DT_301	1D	61	E7	BLINK*, FarmCPU*, GLM*, MLMM*, SUPER*
			E8	BLINK*, FarmCPU*, GLM*, MLMM*, SUPER*
Ra_c69221_1167	5A	42	E3	BLINK**, SUPER**, FarmCPU**, MLMM**, MLM**, GLM*
RAC875_rep_c116515_181	3B	71	E8	MLM**, GLM**, SUPER*, MLMM*, FarmCPU*, BLINK*
RAC875_c14309_317	6B	76	E1	BLINK*, FarmCPU*, MLMM*, SUPER*
RAC875_c23654_214	6B	113	E4	GLM**, BLINK*, FarmCPU*, MLMM*, SUPER*
Excalibur_rep_c69522_83	1B	171	E3	BLINK**, SUPER**, FarmCPU**, MLMM**, GLM**, MLM*
Kukri_rep_c103893_875	2B	65	E5	BLINK**, FarmCPU**, MLMM*, SUPER*, GLM*, MLM*

*Chr, chromosome; Pos, position (cM); Env, environments; E1, Estonia collected in 2019; E2, Estonia collected in 2020; E3, Lithuania; E4, Sweden; E5, Denmark; E6, Estonia 2 years combined; E7, Estonia 2 years and Lithuania combined; E8, all environments combined.*

Model significance: * −log10 (*P*) > 3; ** −log10 (*P*) > 4.

### GWAS for PM

**FIGURE 4:**
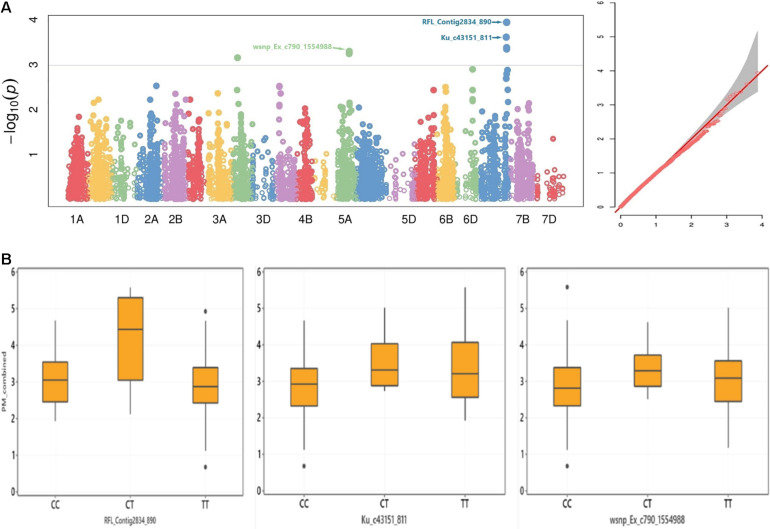
**(A)** Manhattan (left) and Q–Q (right) plots for GWAS result of adult-plant resistance response to PM and **(B)** box plots for STB resistance values grouped by alleles of the SNP markers *RFL_Contig2834_890*, *Ku_c43151_811*, and *wsnp_Ex_c790_1554988*.

The marker *RFL_Contig2834_890* on chromosome 7A had a multi-environment significant MTAs with PM mean scores at *P* < 0.001 (Table 3). This SNP marker alleles had an apparent effect on the distribution of mean values combined from five trials (Figures 4A,B). The SNP markers *wsnp_Ex_c790_1554988* and *Kukri_c6266_260* on chromosome 5A positioned at 98 and 97 cM, respectively, had a significant association with PM from Lithuania (2020), Lithuania 2 years combined, Lithuania 2 years and Estonia 1 year combined, Sweden and Denmark combined, as well as with the all five environments combined mean values. The marker *wsnp_Ex_c790_1554988* had a significant association with noticeable PM disease score effect across its alleles (Figures 4A,B). Genotypes carried the thiamine allele of the marker *Ku_c43151_811* was notably susceptible for PM, while the reverse was true on the SNP marker *RFL_Contig2834_890* (Figure 4B). The SNP marker *BobWhite_c15773_166* on chromosome 2A had a significant MTA with Lithuania (2019), Sweden explaining 19% from the total phenotypic variance (*R*^2^), Denmark and Sweden combined (*R*^2^ = 19.7%), and overall combined PM mean values. The SNP marker *wsnp_Ex_c14340_22315611* on chromosome 3A was the other stable QTN showing significant association with PM scores from Lithuania, Sweden, Denmark, and Lithuania and Estonia combined mean values. A total of 36 SNP markers on chromosome 3A (including the multi-environment QTN, *wsnp_Ex_c14340_22315611*) positioned within 3-cM interval (88–91 cM) exhibited a significant MTAs with PM scores from Lithuania trial conducted in 2019 (Supplementary Table 10 and Supplementary Figure 5B). Chromosomes such as 2D and 7B were the other sources of multi-environment stable QTNs, while 2A, 4B, and 6B were the highly significant multimodel environment-specific QTNs for PM (Table 3).

**TABLE 3:** Summary of GWAS for SNP markers with significant and stable marker trait associations for powdery mildew.

Marker	Chr	Pos	Env	Models
RFL_Contig2834_890	7A	220	E1	GLM**, FarmCPU*, BLINK*
			E2	FarmCPU**, GLM*
			E8	GLM**, FarmCPU*, BLINK*
			E9	BLINK*, FarmCPU*, MLMM*, SUPER*, GLM*, MLM*
			E10	BLINK**, FarmCPU**, MLMM*, SUPER*, MLM*
wsnp_Ex_c790_1554988	5A	98	E3	MLMM**, SUPER**, GLM**, BLINK*, FarmCPU*
			E8	SUPER*, MLMM*
			E9	MLMM*, SUPER*, GLM*, MLM*
			E10	GLM**, SUPER*, MLMM*
Kukri_c6266_260	5A	97	E6	MLMM**, SUPER**, GLM**, BLINK*, FarmCPU*, MLM*
			E9	SUPER*, MLMM*
BobWhite_c15773_166	2A	144	E2	FarmCPU**
			E4	BLINK**, FarmCPU**, MLMM**, SUPER**, GLM**, MLM**
			E6	MLM**, GLM**
			E10	FarmCPU**
wsnp_Ex_c14340_22315611	3A	89	E2	MLMM**, SUPER**, GLM**, MLM*
			E4	GLM*
			E5	GLM*
			E8	GLM**, FarmCPU*, BLINK*
Excalibur_c15048_488	2D	38	E1	GLM*
			E5	MLM*
			E7	BLINK*, FarmCPU*, MLMM*, SUPER*, GLM*
			E8	GLM*
			E9	GLM**, FarmCPU*, BLINK*
RAC875_c8145_1201	7B	134	E2	FarmCPU**
			E8	MLM*, SUPER*, MLMM*
			E9	MLMM**, SUPER**, GLM**, MLM*, BLINK*, FarmCPU*
wsnp_Ex_c12618_20079758	6B	56	E4	BLINK**, FarmCPU**, MLMM**, SUPER**, GLM**, MLM**
RAC875_c1357_860	4B	75	E5	MLMM**, SUPER**, FarmCPU**
RAC875_c1742_2710	7B	83	E5	MLMM**, SUPER**, MLM**, GLM*
Excalibur_c72359_56	2A	150	E6	BLINK**, FarmCPU**, MLMM**, SUPER**, GLM**, MLM*

*Chr, chromosome; Pos, position (cM); Env, environments; E1, Estonia; E2, Lithuania collected in 2019; E3, Lithuania collected in 2020; E4, Sweden; E5, Denmark; E6, Sweden and Denmark combined; E7, Lithuania 2 years combined; E8, Lithuania and Estonia collected in 2019 combined; E9, Lithuania 2 years and Estonia combined; E10, all environments combined.*

Model significance: *−log10 (*P*) > 3; ** −log10 (*P*) > 4.

### Genomic Prediction

**FIGURE 5:**
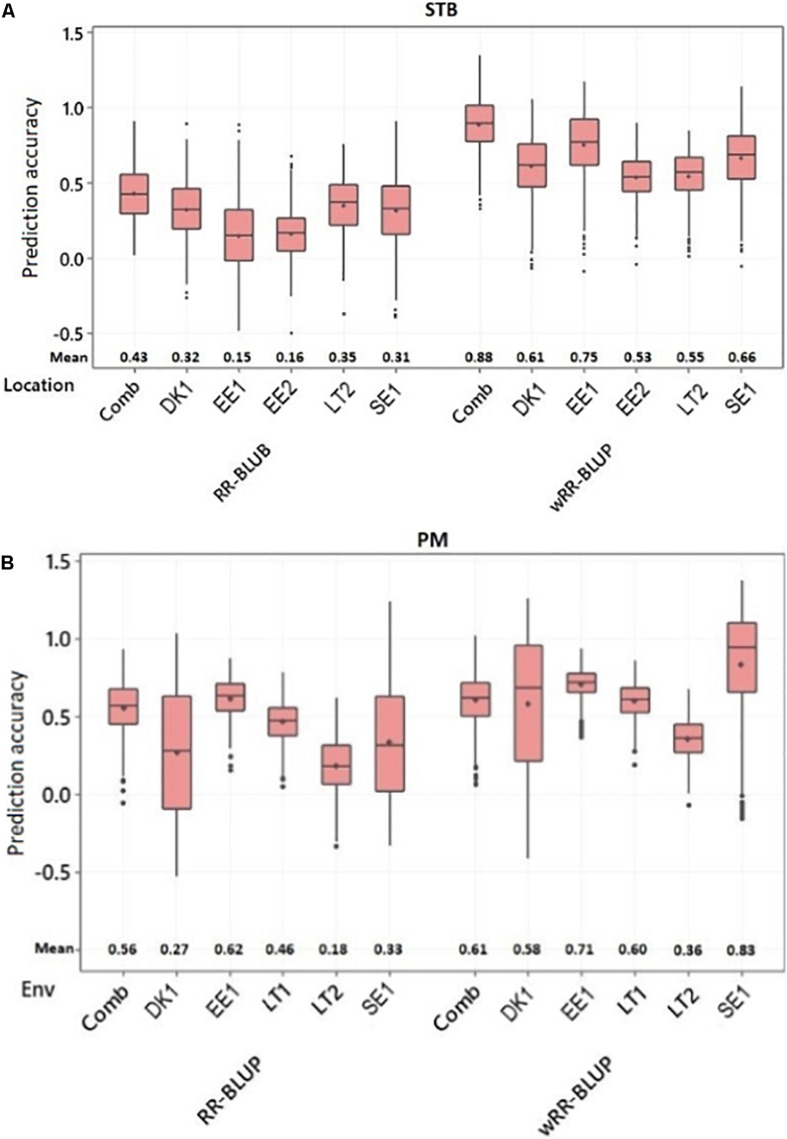
Box plot for the genomic prediction accuracy of the ridge regression BLUP (RR-BLUP) and weighted RR BLUP (wRR-BLUP) models for adult-plant resistance to **(A)** STB and **(B)** PM. Comb, combined; EE, Estonia; LT, Lithuania; SE, Sweden; DK, Denmark; Years: 1, 2018-2019; 2, 2019-2020.

Two different models were applied to predict the genomic breeding values of individual genotypes. GP accuracy was tested through cross-validation using 500 randomly generated datasets from 80 and 20% training and prediction sets, respectively. The first approach was based on the RR-BLUP model in which all SNP markers were fitted as a random effect. With this model, the STB disease score’s GP accuracy was ranged between 0.15 and 0.43. The Estonia trial conducted in 2019 scored the lowest GP accuracy (0.15), while the five environments combined BLUEs mean values scored the highest GP accuracy (0.43) (Figure 5A). For PM disease resistance, the Estonia trial had the highest GP accuracy with 0.62, while the lowest from the Lithuania trial conducted in 2020 with only 0.18 (Figure 5B). The wRR-BLUP was the other utilized model in which the five topmost significant markers detected during the GWAS analysis were fitted as fixed effects. The SNP markers used in this model are given in Supplementary Table 19. This model significantly improves the GP prediction accuracy of the previous model for both disease traits, signifying large effects from these QTLs. For instance, the GP accuracy of STB APR trials conducted in Sweden and Denmark improved from 0.31 and 0.32 to 0.66 and 0.61, respectively (Figure 4A). Likewise, the GP accuracy for PM disease scores conducted in Sweden and Estonia increased from 0.33 and 0.46 to 0.83 and 0.60, respectively (Figure 5B).

## Discussion

This study evaluated 175 genetically diverse winter wheat genotypes from the NordGen genebank to detect QTLs and estimate genomic breeding values of genotypes for adult-plant disease resistance for STB and PM. These two foliar diseases are regarded as two major biotic threats for European winter wheat production. The panel integrates landraces and cultivars originated from Sweden, Denmark, Finland, and Norway, representing the previous century winter wheat breeding practices in the region (Odilbekov et al., 2019a). Genotypes demonstrated a wide range of genetic variation for STB and PM disease resistance response in adult plants scored across five environments. The presence of medium to high range of repeatability scored across studied environments coupled with the existing higher genetic variation designates the suitability of the phenotypic data for GWAS and GP analysis.

### GWAS and GP for STB

The panel was previously tested for seedling resistance to STB with artificial inoculation in Biotron greenhouse chamber, and genotypes had a significant genetic variation for STB resistance response (Odilbekov et al., 2019a). In this study, a highly significant genetic variation was observed for STB disease APR across the five studied environments tested under field conditions. In addition, highly significant G × E interaction was recorded for the environments combined analysis, indicating the unequal disease pressure across locations and years, which is a widely occurring phenomenon in previous studies (Dreisigacker et al., 2015; Muqaddasi et al., 2019). The quantitatively inherited genetic factors for STB are generally prone for G × E interactions (Schilly et al., 2011). Nonetheless, the recorded higher genetic variance coupled with the medium to high environment-specific repeatability could make the phenotypic data convenient for GWAS and GP analysis.

In addition to the renowned *stb* genes, several QTLs have been involved for resistance to STB in wheat with moderate-to-small effects (Brown et al., 2015). All the seedling stage QTLs identified previously by Odilbekov et al. (2019a), except those on chromosomes 1A and 3A, were also detected as stable and environment-specific QTNs in this study attesting their potential to be the source of genes for STB resistance at all plant stages. He et al. (2020) discovered an APR QTL against STB in common wheat line Murga on chromosome 3B, while Odilbekov et al. (2019b) reported a seedling-resistant QTL on chromosome arm 1BL (96–99 cM). Similarly, significant SNPs were detected in this study on 1BL from Sweden (95 cM), Denmark (155–159 cM), and Lithuania (171 cM) trials. This study detected stable as well as environment-specific QTLs for STB APR. The marker *wsnp_Ex_c33012_41567026* on the long arm of chromosome 4A, positioned at 739.52 MB [according to the International Wheat Genome Sequencing Consortium (2018)], was one of the stable QTNs detected in this study. This chromosome region is the source of race-specific resistance genes, including *Stb7* (McCartney et al., 2003) and *Stb12* (Chartrain et al., 2005). However, due to the different marker tools applied, it was difficult to put this SNP marker’s exact position related to the genes mentioned earlier. Notwithstanding, Muqaddasi et al. (2019) reported an SNP marker *wsnp_JD_c27162_22206547* (664.14 MB) for adult-plant STB resistance in European Winter Wheat, which is found close to the currently detected marker. Similarly, Riaz et al. (2020) detected QTLs on chromosome arm 4AL (*BS00040648_51*, 594 MB) for adult-plant STB resistance using an eight-founder MAGIC winter wheat population. The other stable marker detected in this study was *BS00021714_51* (78 cM), according to Wang et al. (2014b) on the long arm of chromosome 1A, which is nearby to the SNP marker *wsnp_Ex_rep_c109742_92411838* (81 cM) discovered previously by Muqaddasi et al. (2019). Riaz et al. (2020) also found a QTL on this chromosome region (70–78 cM) for STB resistance in winter wheat adult plants in the field. Chromosome 5A was the other region comprising a lot of SNP markers with stable and environment-specific highly significant QTNs for STB resistance including *Kukri_c63163_141* that explained 8.0% from the total phenotypic variance. Chromosome arm 5AL is a region in which *stb17* gene was discovered previously in adult plants of synthetic hexaploid wheat (Ghaffary et al., 2012), which is nearby (62 cM) to our finding (63 cM). Similarly, Muqaddasi et al. (2019) reported multi-QTNs on chromosome 5A (48.1–90.4 cM) for STB resistance to adult plants of European Winter Wheat. Dreisigacker et al. (2015) detected a QTL (29.4–36.9 cM) on chromosome arm 5AL for APR to STB in spring bread wheat. This study identified a stable QTN on chromosome 6A (*wsnp_Ku_c38451_47086066*, 79 cM) in which the race specific resistance gene *stb15* was detected previously (Arraiano et al., 2007). This gene is commonly found in European winter wheat cultivars, but its resistance was ratified in the seedling stage. However, Miedaner et al. (2013) detected a QTL on chromosome 6AS (14.2 cM) for STB APR in 1055 elite wheat hybrids and their 87 parental lines although quite far from the QTN detected in this study. Moreover, the current study detected other stable as well as environment-specific QTLs on chromosome arm regions including 1DS, 2BS, 3BL, 3BS, and 6BL in which most of them were reported previously by others including Riaz et al. (2020), but the 1DS and 3BL QTLs could be potentially newly discovered in this study for STB APR in wheat.

For the past two decades, GP has been effectively used as a powerful tool to select favorable genetic material for traits of interest based on their dense genome-wide markers (Meuwissen et al., 2001; Mirdita et al., 2015; Crossa et al., 2017). GP could even be more feasible for traits like STB in which several QTLs with minor-to-moderate effect are involved in plant resistance (Brown et al., 2015). Besides, most known *stb* genes are one or few race-specific pathogen (Brown et al., 2015) and lack the broad-spectrum application. Hence, incorporating several QTLs is the most sustainable approach in developing breeding lines with resistance to the pathogen. Since the current panel comprises both landraces and historical cultivars, GWAS detected several QTLs with minor-to-moderate effect that suggested the GP method could be more appropriate to account them all and predict lines based on their overall marker effect. In this study, two GP models (i.e., RR-BLUP and wRR-BLUP) were implemented to predict accessions’ genomic breeding values. The GP accuracy of STB resistance with RR-BLUP model was low to moderate across environments with a range of 15–35%. Considering the highly diverse genetic composition, smaller numbers of genotypes, and marker density used to train the model, the recorded GP accuracy could be promising. This model assumes that all markers share a common variance, and their effects are shrunken toward zero (Lorenz et al., 2011). This can lead to underestimation of variances on QTLs with major to moderate effects. To overcome this problem, a wRR-BLUP was implemented in which QTLs with major effect could be fitted as fixed effects (Spindel et al., 2016). In this study, the GP accuracy increased significantly when the five topmost significant SNP markers for STB resistance were fitted as fixed effect. The wRR-BLUP improved the GP accuracy and ranged 53–75% across environments. This outcome is in agreement with previous studies (Arruda et al., 2016; Spindel et al., 2016; Herter et al., 2019).

However, it is important to note that the prediction accuracies of wRR-BLUP could be overestimated, since the significant markers from the GWAS fitted as fixed effects were obtained from the complete dataset (100%) but not from each of the simulated 500 training set (TS). For unbiased estimates in wRR-BLUP, separate GWAS must be conducted for each of the 500 TSs to detect significant SNPs fitted in the wRR-BLUP model as fixed effects. Unfortunately, this is highly computationally demanding (Gaikpa et al., 2021) because it will require 500 runs each for the GWAS, TS, and test sets. In a practical GP breeding program, one TS is usually involved instead of the 500 pseudo-TS simulated in our study. Besides, in practical breeding, we aim to identify QTLs in certain populations and validate these QTLs and markers designed for marker-assisted selection. The markers developed from stable QTLs can be used to screen across diverse populations, which may be connected at different levels to the original population(s) used to identify the QTLs for the markers. In this case, separate GWAS or QTL analyses are not conducted for each breeding population once markers are available. Therefore, the identification of markers in the entire dataset by GWAS for wRR-BLUP in our simulation study can provide an insight into the prospect of genomic selection using a subset of SNPs identified by GWAS for disease resistance breeding in wheat (Herter et al., 2019). An independent training and validation set might result in unbiased estimates of the prediction accuracy.

### GWAS and GP for PM

The panel was tested in field conditions for PM resistance in five environments. The results indicated a highly significant variation in resistance response toward the pathogen (Table 1). A significant G × E interaction was observed for PM response, but a higher broad-sense heritability (69%) was recorded from environment-combined analysis assuring the quality of the phenotypic data and its suitability for detecting trustworthy QTLs and predicting the genomic estimated breeding values (GEBVs).

The wheat plant resistance to PM is inherited qualitatively by the major *Pm* genes and quantitatively by small-to-moderate effect genes (Alam et al., 2011). The major *Pm* genes are generally race specific and effective against particular PM isolates that makes them last for a short duration due to the unpredicted and frequent change of the pathogen population (Marone et al., 2013). Subsequently, adult-plant resistance, also called slow mildewing, controlled by several genes (polygenes/QTLs), is introduced as the source for durable PM resistance in wheat (Liang et al., 2006). The current study detected several stable and environment-specific QTLs for PM resistance in adult winter wheat. The marker *RFL_Contig2834_890* (722.30 MB) on chromosome arm 7AL, where the *Pm1* gene was detected previously (Hartl et al., 1995; Neu et al., 2002), had a stable significant association with PM APR. Generally, chromosome 7A is a source of several *Pm* genes as well as other singleton and meta-QTLs (Muranty et al., 2010; Marone et al., 2013). The SNP marker *BobWhite_c15773_166* (752.09 MB) on the chromosome arm 2AL was the other source for stable QTN discovered in all environments for PM APR. The chromosome arm 2AL is the other gene-rich region for PM resistance including the *Pm4a*, *b*, and *d* alleles detected on the chromosome homoeologous group two, which was initially introgressed from other progenitors species of wheat (Ma et al., 1994; Schmolke et al., 2012), and other QTLs (Marone et al., 2013; Nordestgaard et al., 2020). In this study, highly significant multi-QTNs were detected on the long arm of chromosome 5A (63–98, 115 cM) across environments for PM adult-plants resistance. Chromosome 5A is the other gene-rich region for PM resistance for wheat acquired through translocation and introgression (Marone et al., 2013). This study detected a stable QTL on chromosome arm 2AL (144–150 cM). Similarly, Yang et al. (2017) detected across environments stable QTL on both chromosome arms 2AL (163.2–168.8 cM) and 5AL (225.1–237.6 cM) for PM resistance in adult plants using doubled haploid populations derived from Chinese wheat cultivars. The SNP marker *wsnp_Ex_c14340_22315611* on chromosome arm 3AL (89 cM) was another stable QTN discovered in this study. Previous studies reported QTLs on this chromosome region nearby to this marker for PM APR (Keller et al., 1999), and for both seedling and adult stage PM resistance positioned on 80.8 and 83.4 cM, respectively (Simeone et al., 2020). Furthermore, this study detected stable and environment-specific QTLs for PM resistance to adult plants including on chromosome arms 2DS, 4BL, 6BL, 7AL, and 7BL. Previous studies reported QTLs on these chromosome regions (Stadlmeier et al., 2019; Simeone et al., 2020), except the QTL discovered on 2DS that could possibly be a newly detected QTL in this study.

Genomic prediction analysis was done with both the RR-BLUP and wRR-BLUP models to estimate the genomic breeding values of individual genotypes for PM APR. The two models predicted higher GP accuracy to PM than STB that could be due to the higher heritability recorded on the earlier (PM) earlier in both single as well as environment-combined analysis. The GP prediction accuracy of PM ranged between 18 and 62% across environments for the RR-BLUP model. The wRR-BLUP improved the GP prediction accuracy, and the score was in a range of 36–83% across the tested environments. In a previous study, Sarinelli et al. (2019) detected a GP accuracy for adult-plant PM resistance in a range of 36–55 and 42–60% with unweighted and weighted RR-BLUP models, respectively, using a varied set of training population sizes for 467 historical United States winter wheat genotypes. Nonetheless, similar to the STB case, the later model could overestimate the GP accuracy due to individuals’ inclusion in both the GWAS and validation sets.

## Conclusion

This study analyzed 175 winter wheat genotypes from NordGen comprising historical landraces and cultivars for GWAS and GP analysis. The phenotypic data indicated a significant variation between genotypes for adult-plant disease resistance response to STB and PM. The GWAS analysis identified various valuable QTNs for both STB and PM APR that could be incorporated in future marker-assisted selection and genomic selection programs. GWAS significantly improved the GP accuracy in predicting the genomic breeding values of genotypes for both STB and PM APR. The result of this study supports the potential of GP in predicting the GEBVs of the vastly available genetically rich landraces by developing appropriate models. The genomic-assisted germplasm selection with superior alleles for disease resistance in wheat could be then integrated into active breeding programs.

## Data Availability Statement

The datasets presented in this study can be found in online repositories. The names of the repository/repositories and accession number(s) can be found in the article/Supplementary Material.

## Author Contributions

AC conceived and designed the study. TH performed field the trials in Sweden. JS and LJ performed the field trials in Denmark. GB and ŽL performed the field trials in Lithuania. RK, BI, and MK performed the field trials in Estonia. AA and DSG performed the data analysis and wrote the first draft of the manuscript. All authors contributed to the data interpretation and approved the final version of this manuscript.

## Conflict of Interest

The authors declare that the research was conducted in the absence of any commercial or financial relationships that could be construed as a potential conflict of interest.

## Footnotes

1 www.nordgen.org
