# Supplementary material for: Genome-Wide Association Analysis and Genomic Prediction for Adult-Plant Resistance to Septoria Tritici Blotch and Powdery Mildew in Winter Wheat
Source: Front Genet. 2021 May 12;12:661742. doi: 10.3389/fgene.2021.661742 (PMC8149967; doi:10.3389/fgene.2021.661742)
Supplement: Supplementary file 1 [file Data_Sheet_1.ZIP › Supplementary files/Supplementary figures.docx]

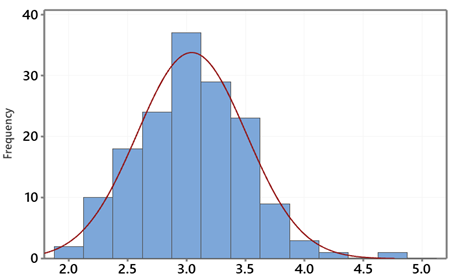

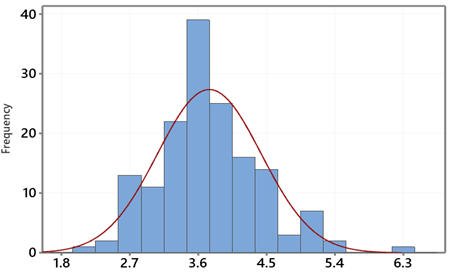


**B**

**A**


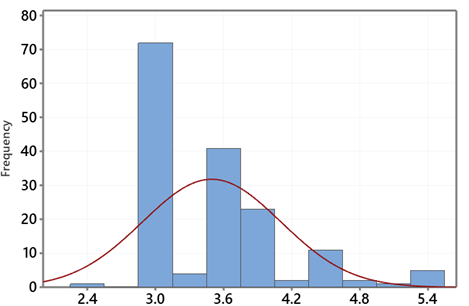

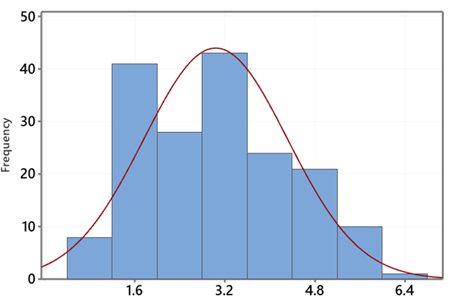


**D**

**C**


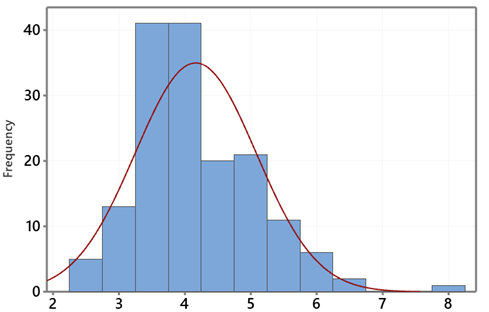


**E**

**Supplementary figure 1**. Frequency distribution of the STB diseases scored for 175 winter wheat genotypes from Estonia collected in the year 2019 (**A**) and the year 2020 (**B**), Lithuania (**C**), Denmark (**D**), and Sweden (**E**).


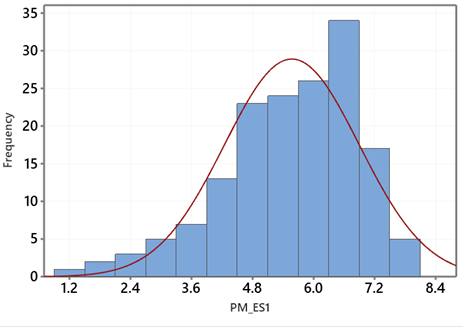

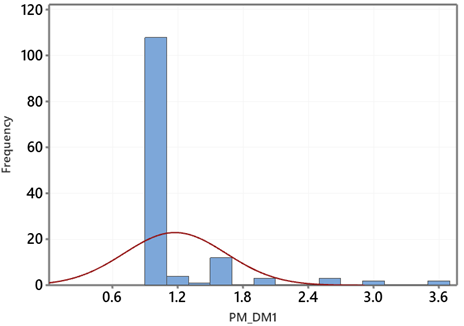


**A**

**D**

**B**


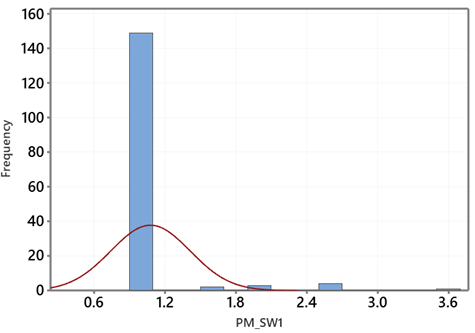

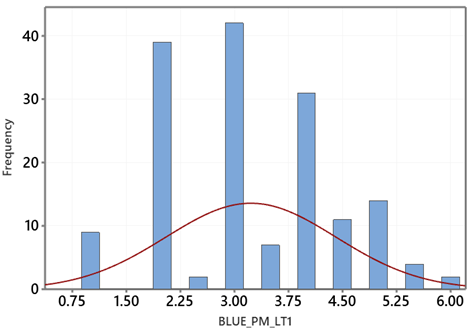


**C**


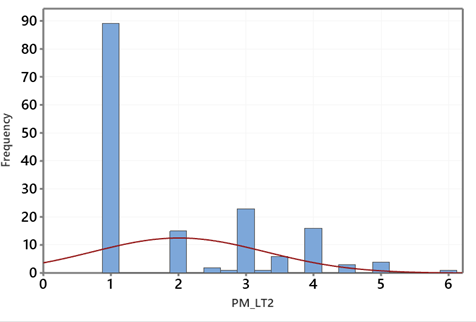


**E**

**Supplementary figure 2**. Frequency distribution of the PM diseases scored from Estonia (**A**), Denmark (**B**), Sweden (**C**), and Lithuania collected in the year 2019 (**D**), and in the year 2020 (**E**).


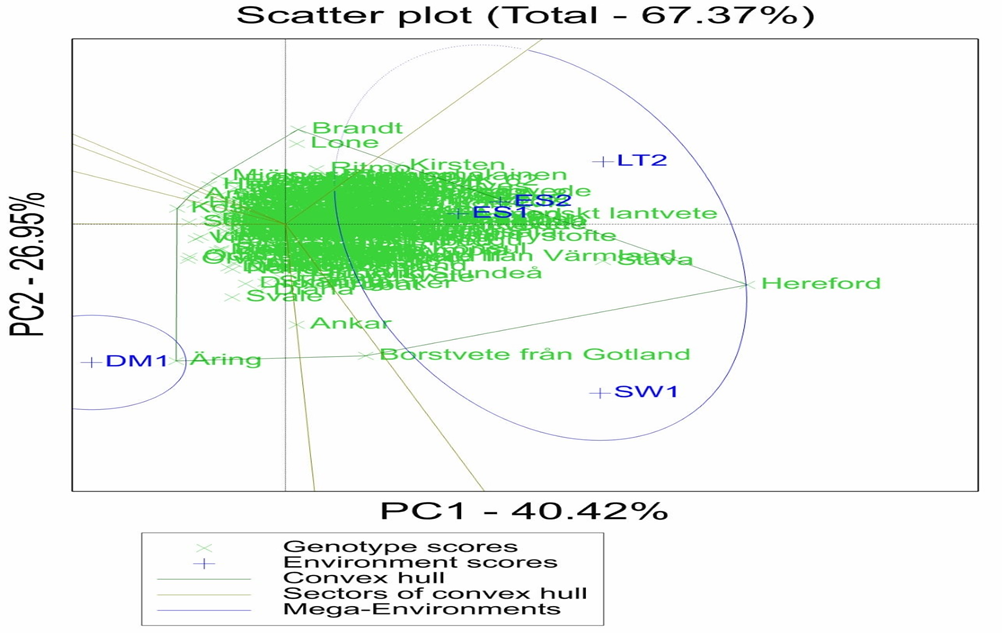


**A**


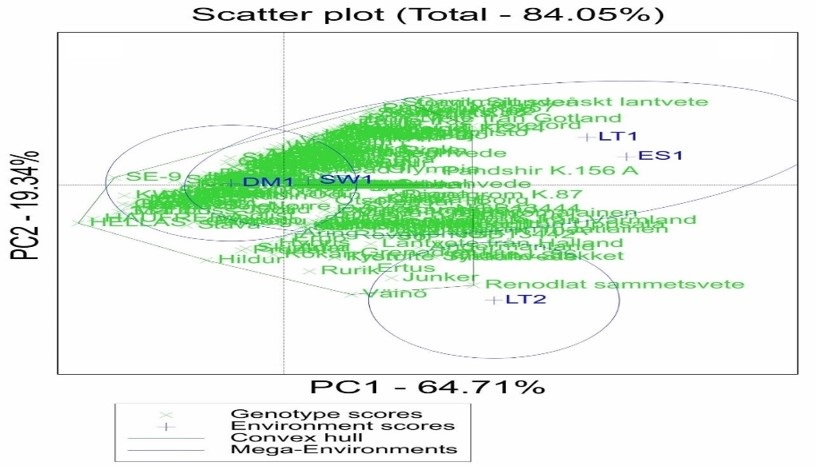


**B**

**Supplementary figure 3**. Genotype plus genotype-by-environment interaction (GGE) biplot result for STB (**A**) and PM (**B**) disease scores from 175 winter wheat genotypes collected in five environments.


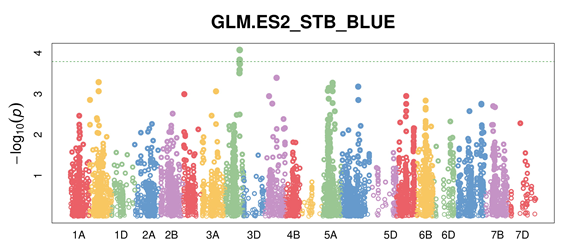

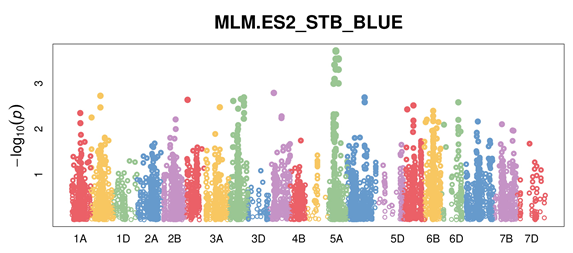

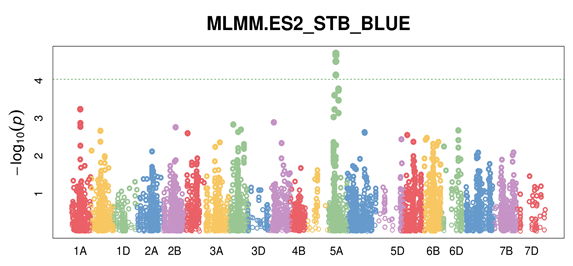


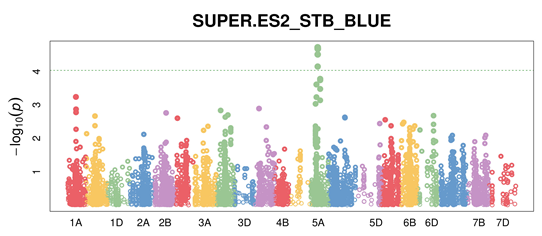

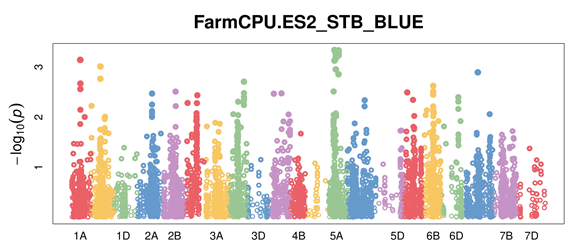

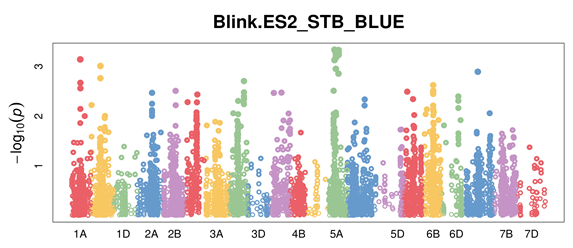


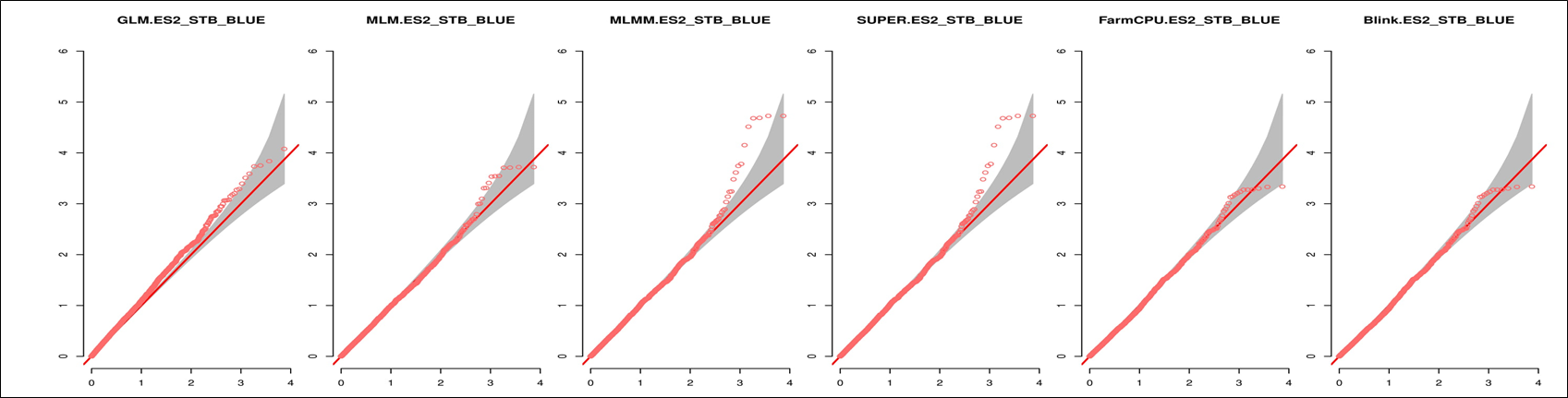


**Supplementary figure 4A**. Manhattan and Q-Q plots of the GWAS result for STB disease score collected in Estonia in 2020. The horizontal dash line indicates the FDR adjusted P-value at 0.01.


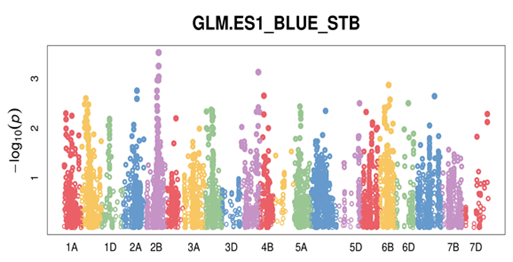

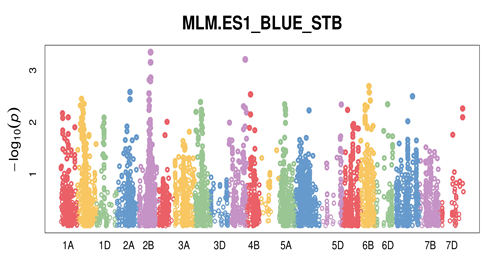

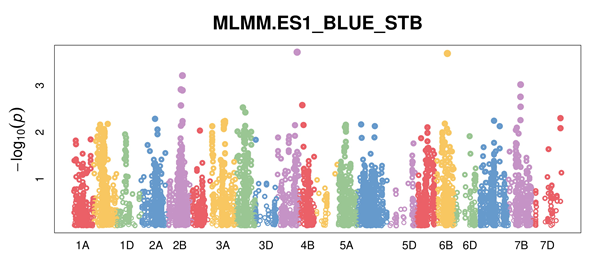


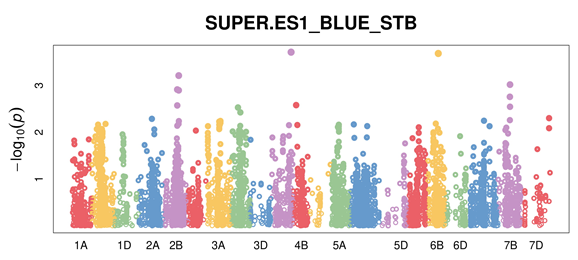

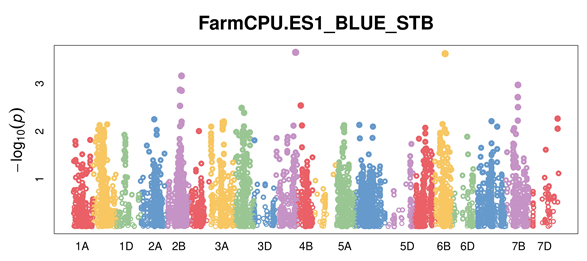

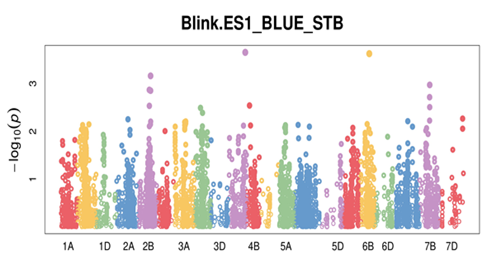


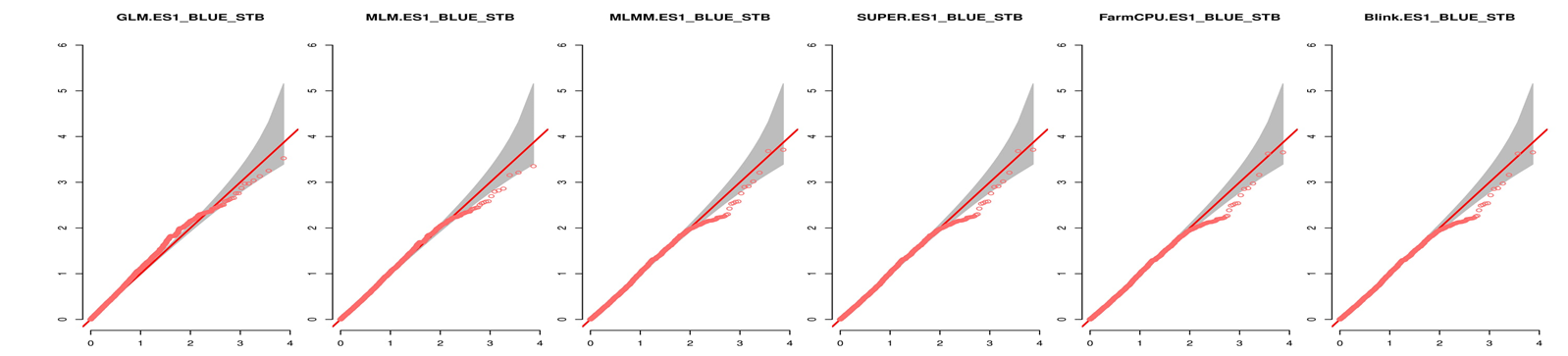


**Supplementary figure 4B**. Manhattan and Q-Q plots of the GWAS result for STB disease scored in Estonia in 2019.


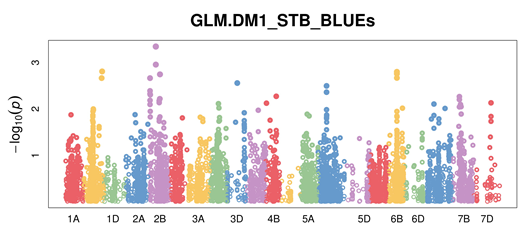

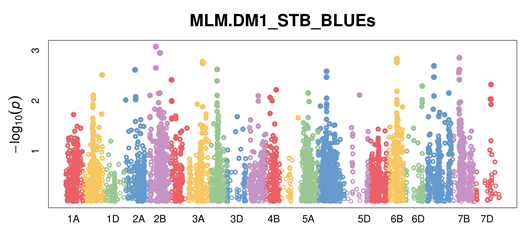

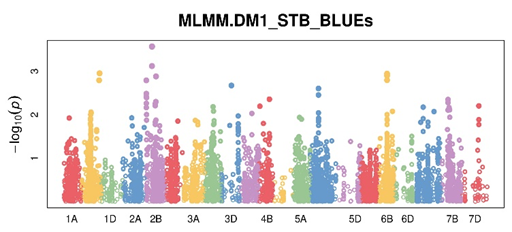


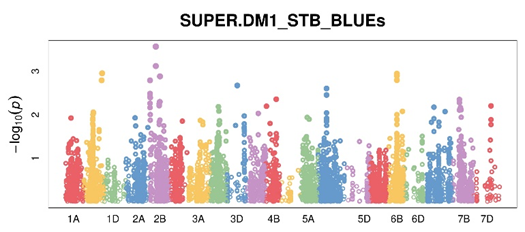

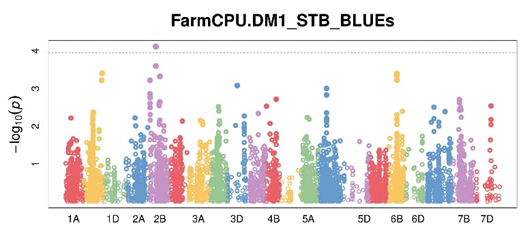

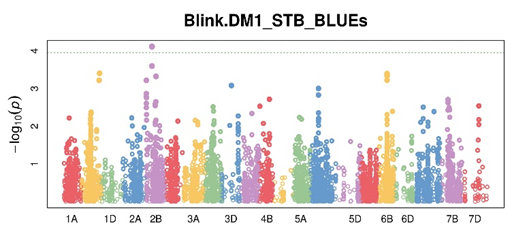


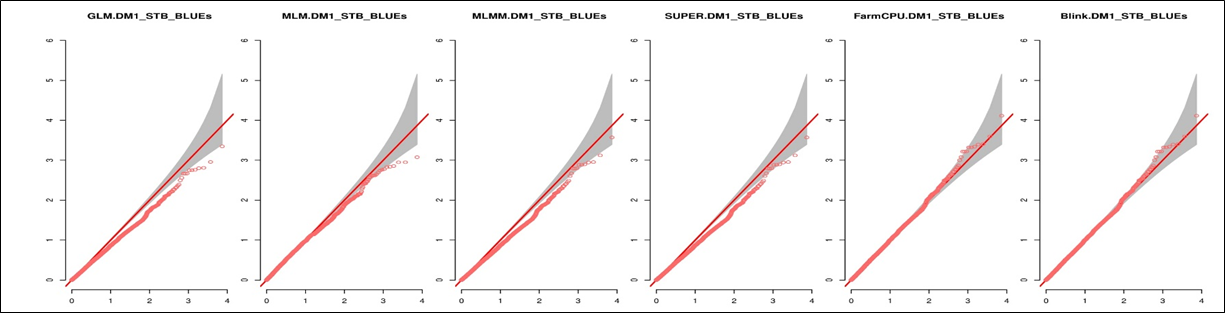


**Supplementary figure 4C**. Manhattan and Q-Q plots of the GWAS result for STB disease scored from Denmark in 2019. The horizontal dash line indicates the FDR adjusted P-value at 0.01.


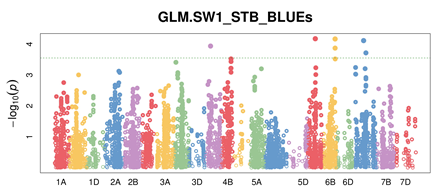

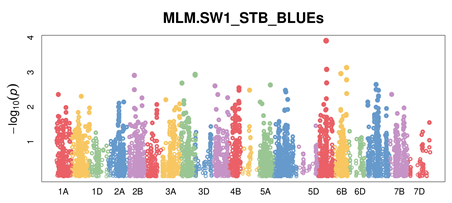

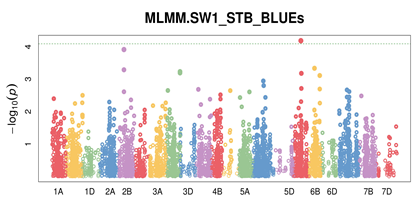


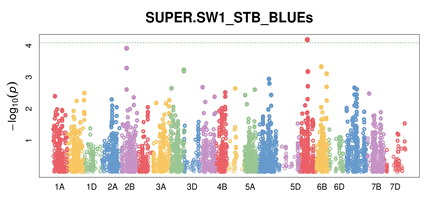

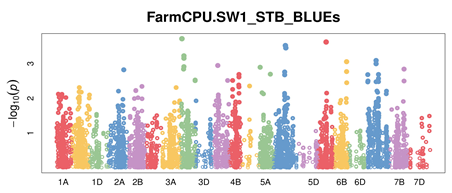

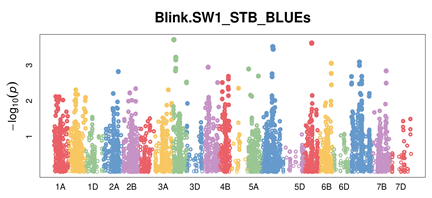


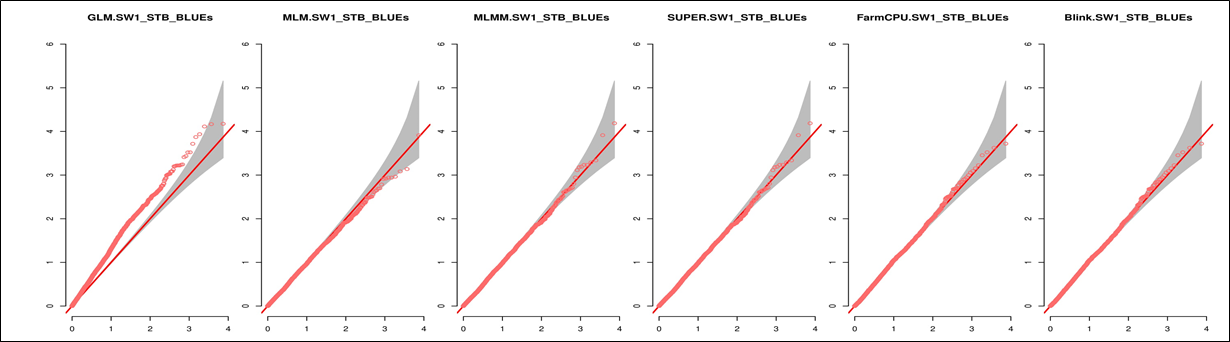


**Supplementary figure 4D**. Manhattan and Q-Q plots of the GWAS result for STB disease score collected from Sweden in 2019. The horizontal dash line indicates the FDR adjusted P-value at 0.01.


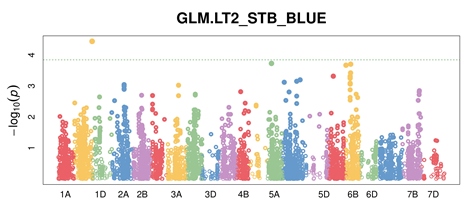

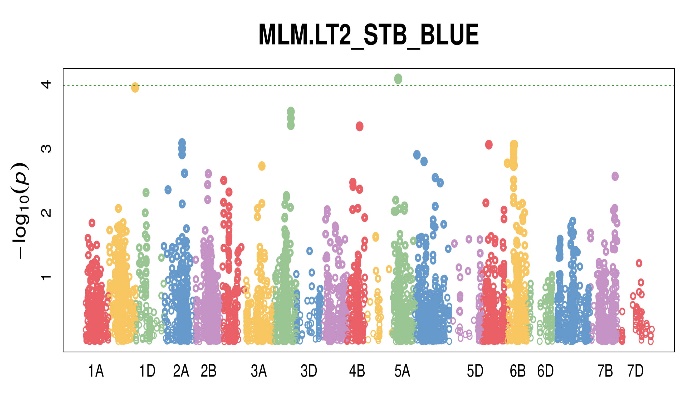

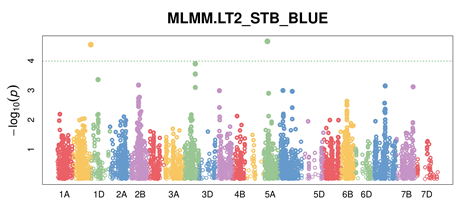


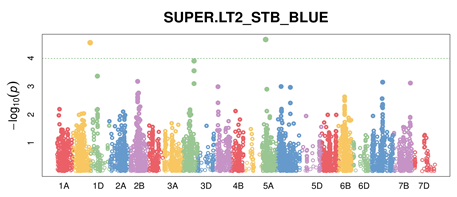

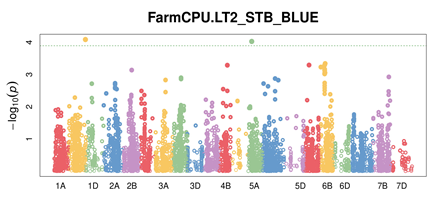

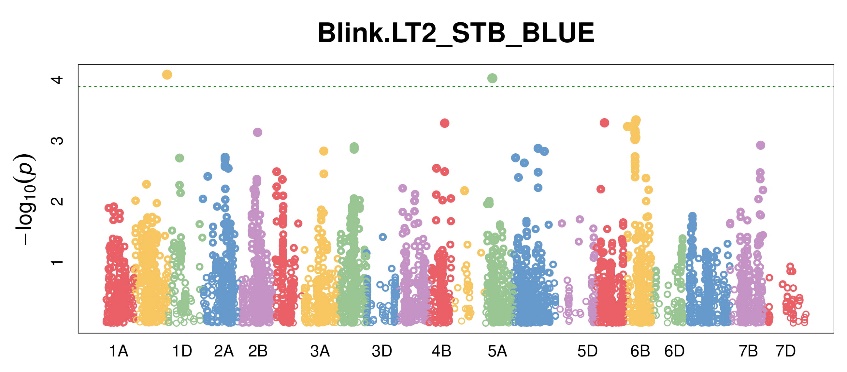


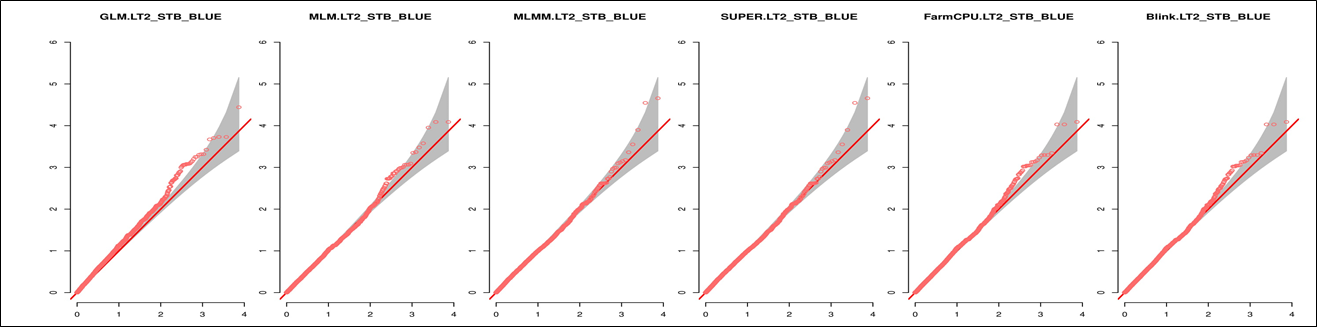


**Supplementary figure 4E**. Manhattan and Q-Q plots of the GWAS result for STB disease scored from Lithuania in 2020. The horizontal dash line indicates the FDR adjusted P-value at 0.01.


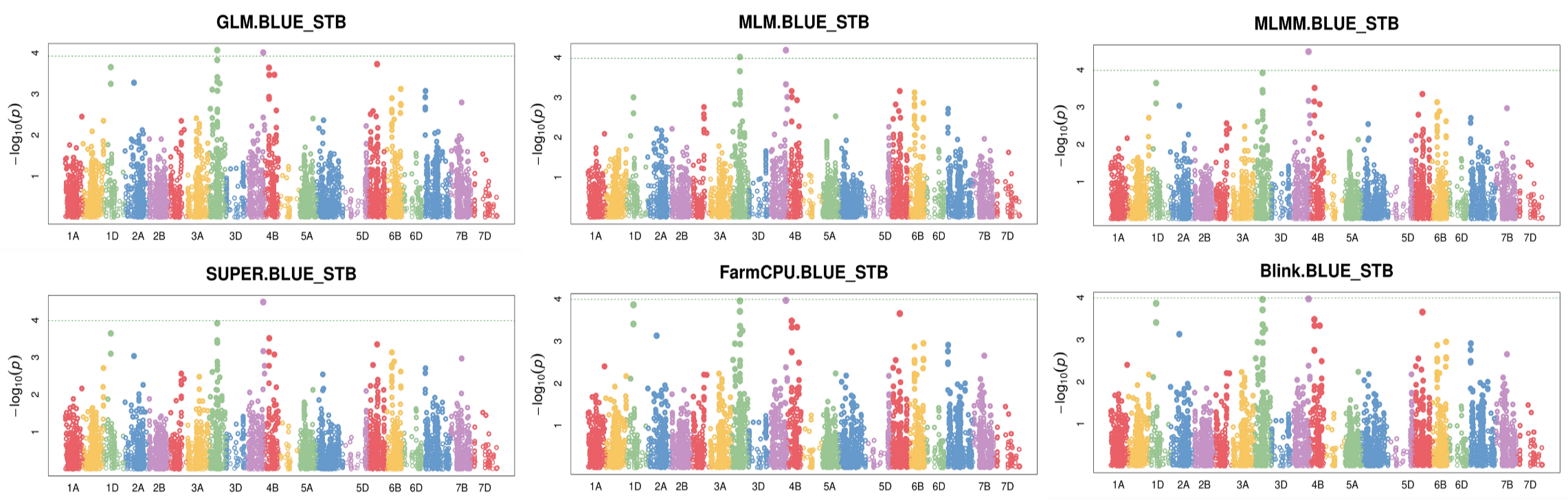


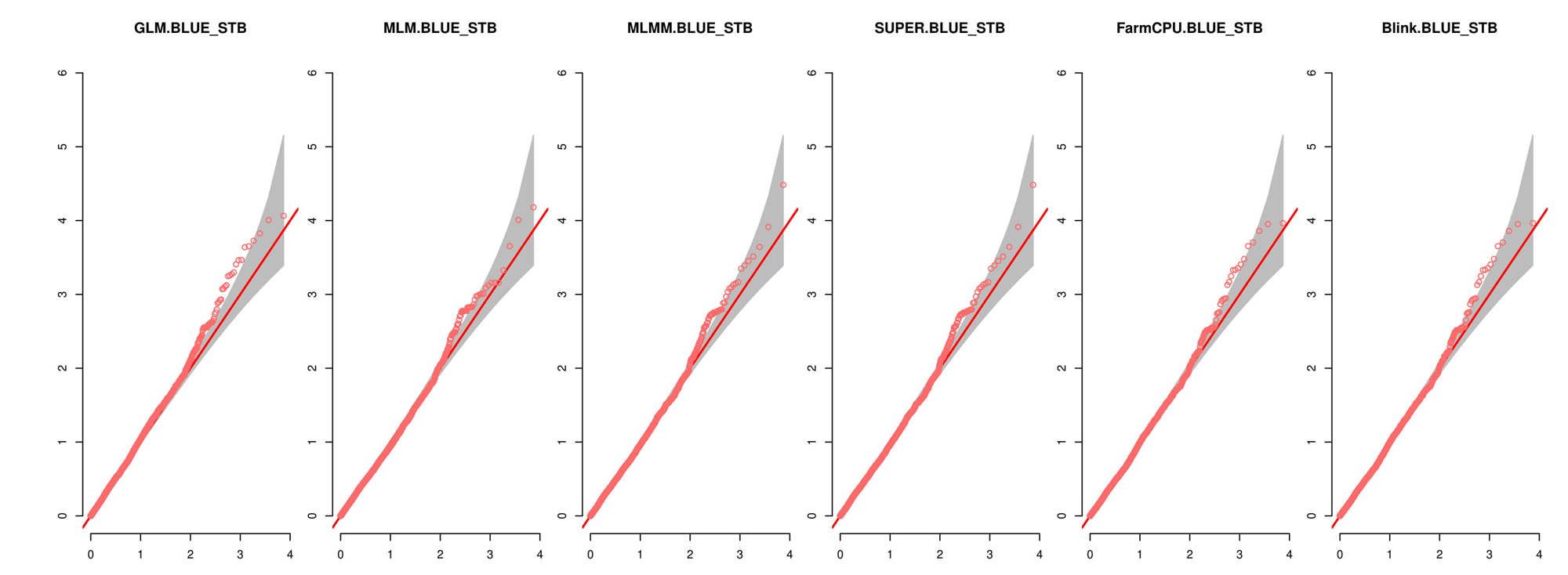


**Supplementary figure 4F**. Manhattan and Q-Q plots of the GWAS result for STB disease score combined from five environments tested at four locations. The horizontal dash line indicates the FDR adjusted P-value at 0.01.


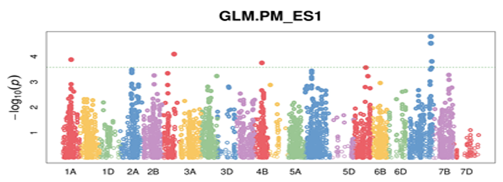

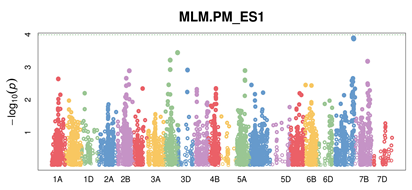

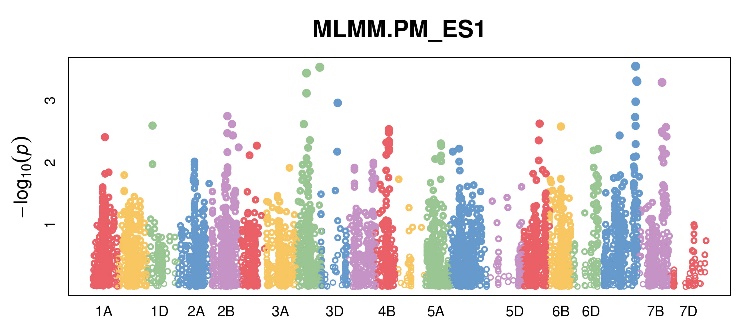


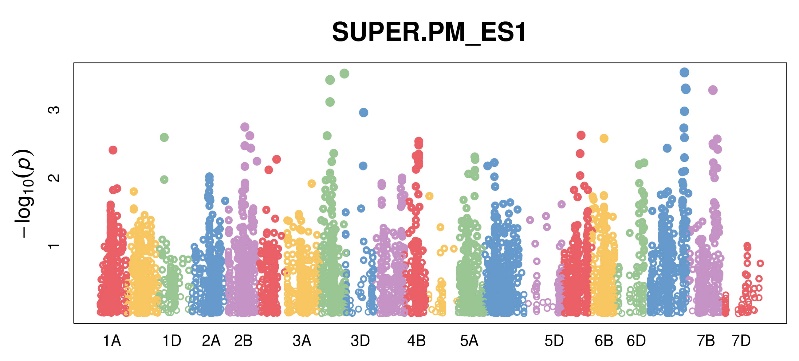

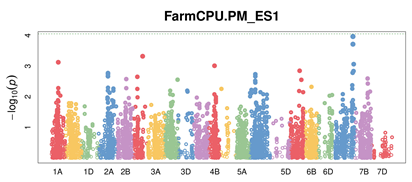

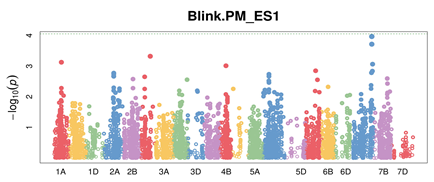


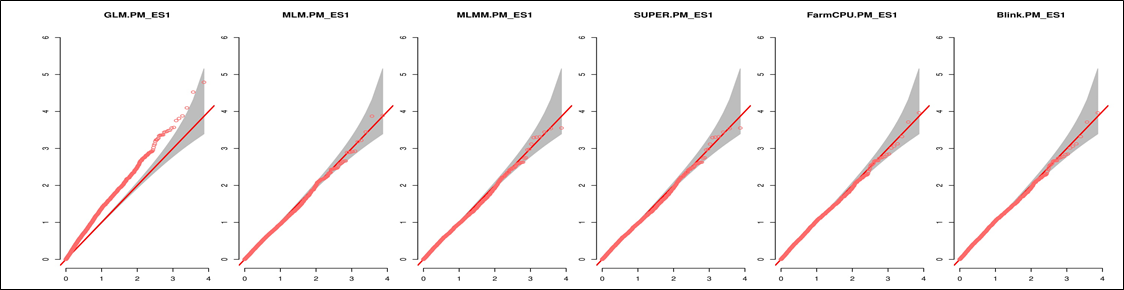


**Supplementary figure 5A**. Manhattan and Q-Q plots of the GWAS result for PM disease score collected from Estonia in 2019. The horizontal dash line indicates the FDR adjusted P-value at 0.01.


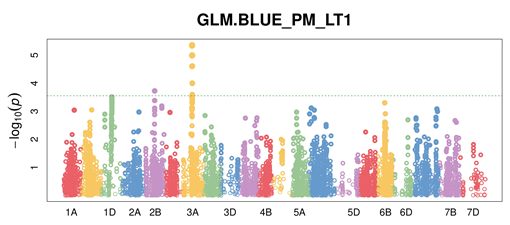

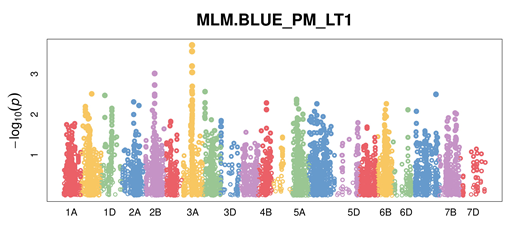

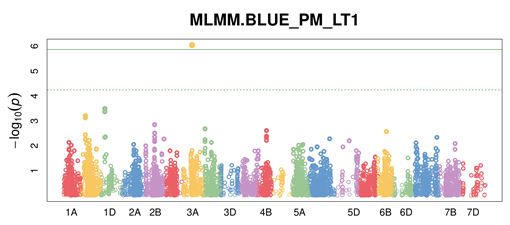


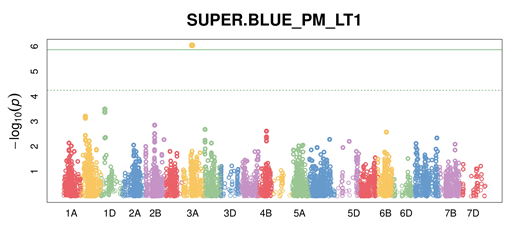

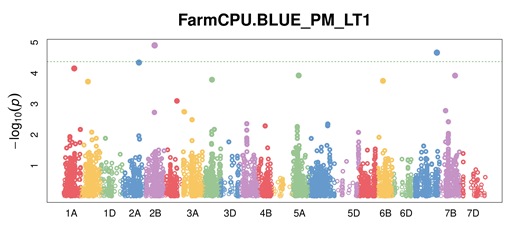

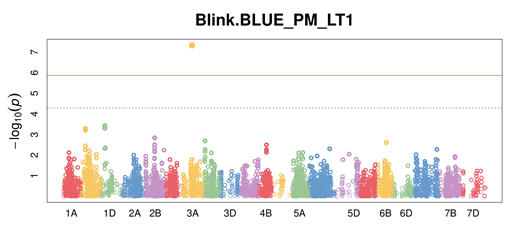


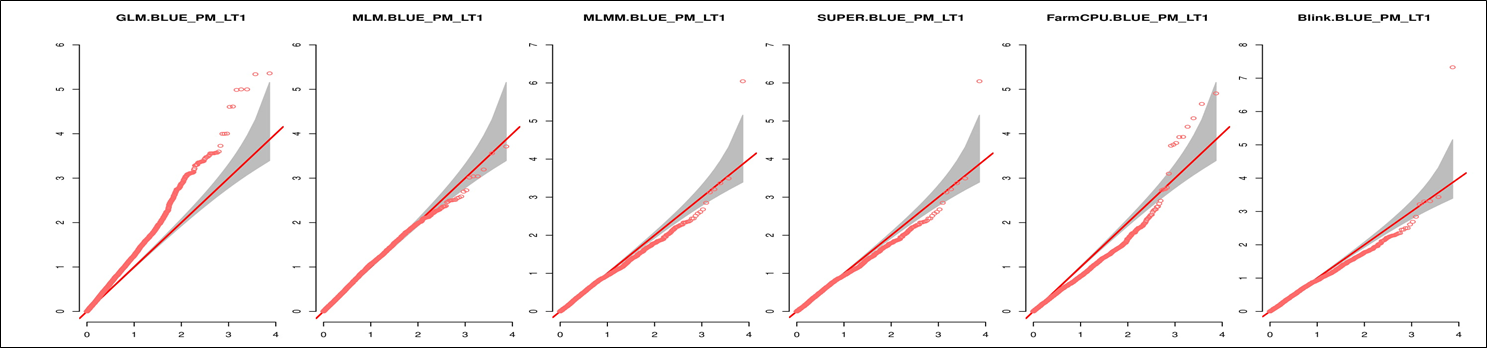


**Supplementary figure 5B**. Manhattan and Q-Q plots of the GWAS result for PM disease score collected from Lithuania in 2019. The horizontal dash and solid lines indicate the FDR adjusted P-value at 0.01 and Bonforreni corrected P-value at 0.05, respectively.


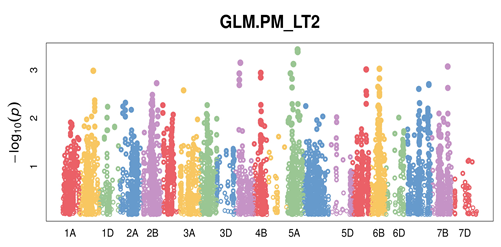

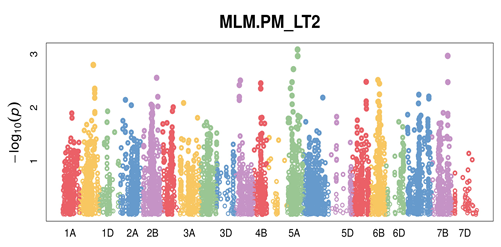

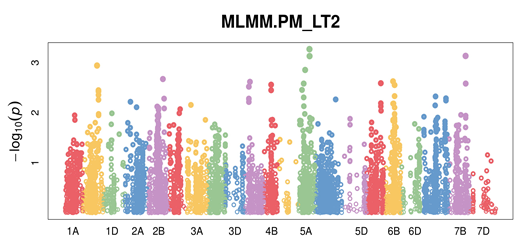


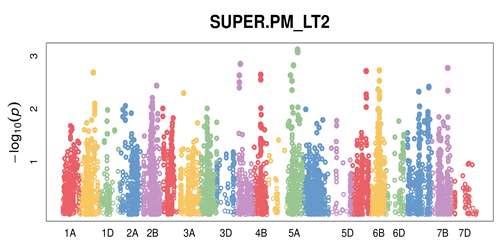

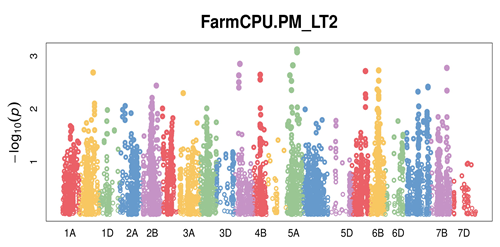

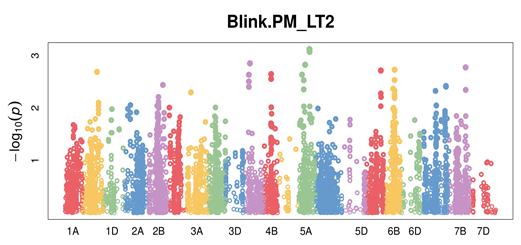


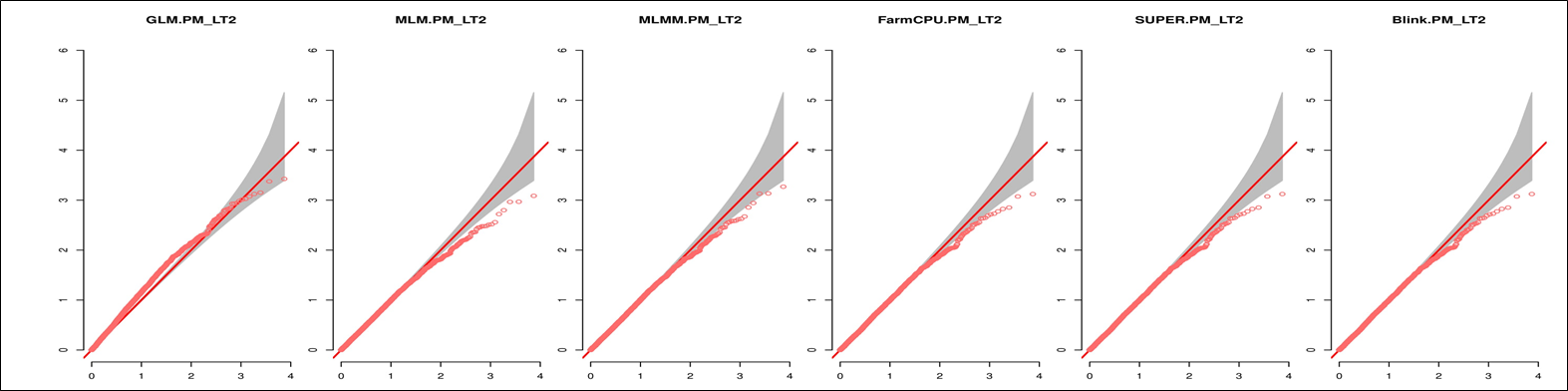


**Supplementary figure 5C**. Manhattan and Q-Q plots of the GWAS result for PM disease score collected from Lithuania in 2020.


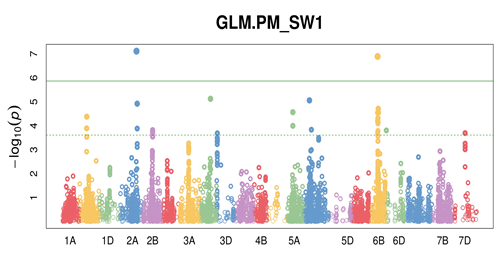

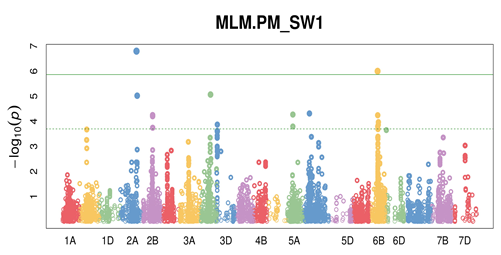

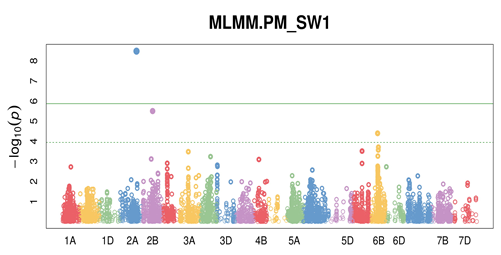


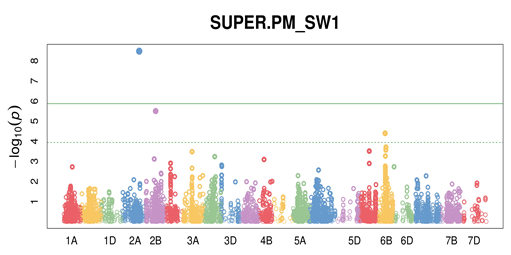

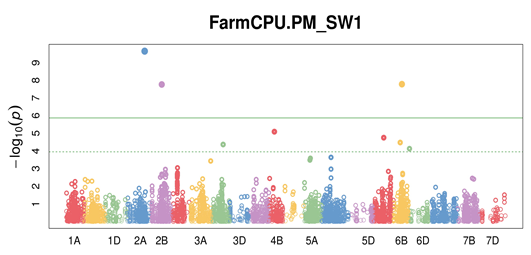

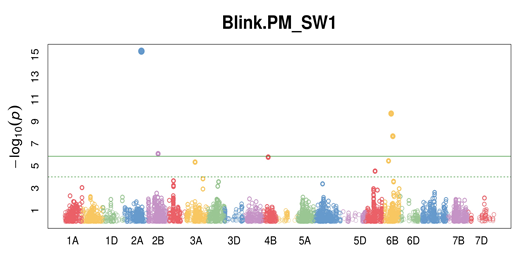


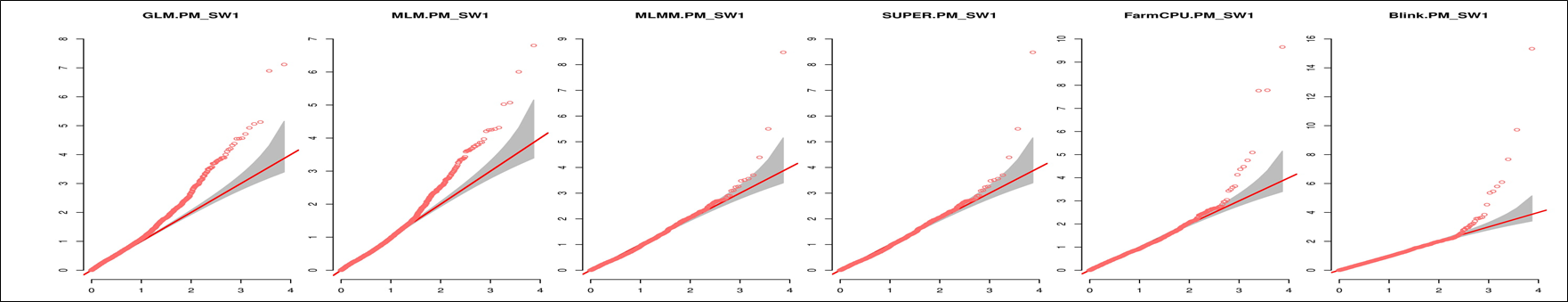


**Supplementary figure 5D**. Manhattan and Q-Q plots of the GWAS result for PM disease score collected from Sweden in 2019. The horizontal dash and solid lines indicate the FDR adjusted P-value at 0.01 and Bonforreni corrected P-value at 0.01, respectively.


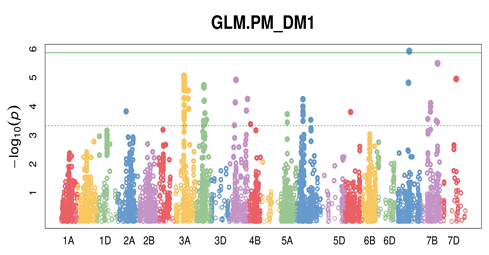

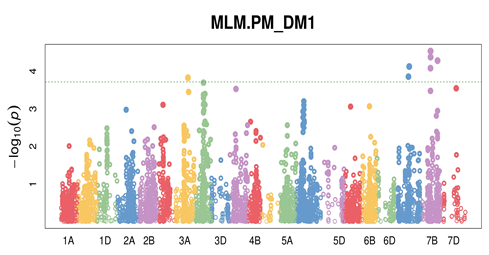

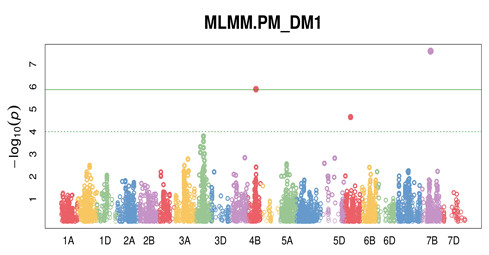


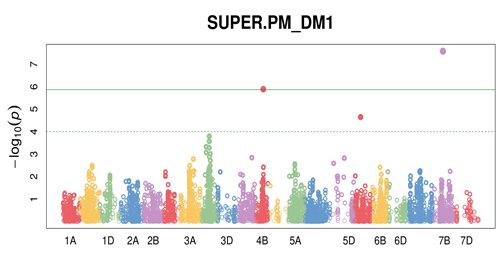

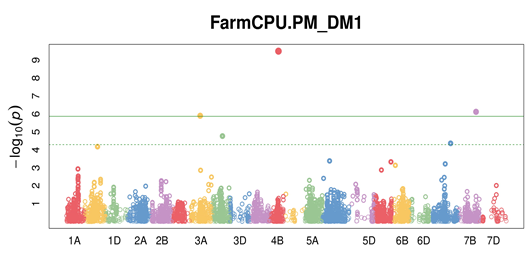

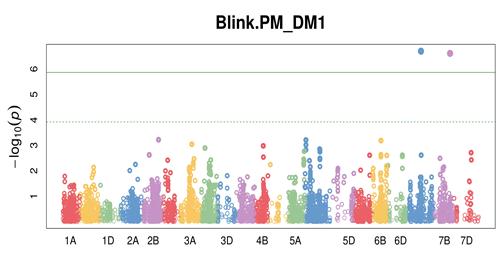


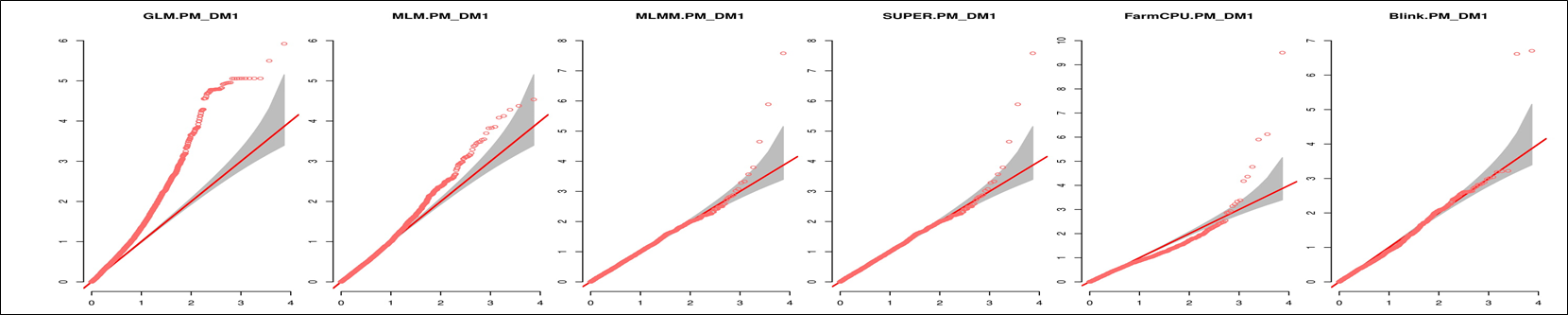


**Supplementary figure 5E**. Manhattan and Q-Q plots of the GWAS result for PM disease score collected from Denmark in 2019. The horizontal dash and solid lines indicate the FDR adjusted P-value at 0.01 and Bonforreni corrected P-value at 0.01, respectively.


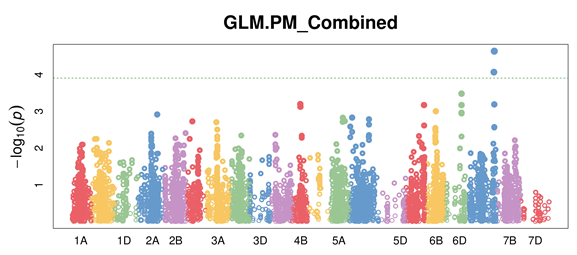

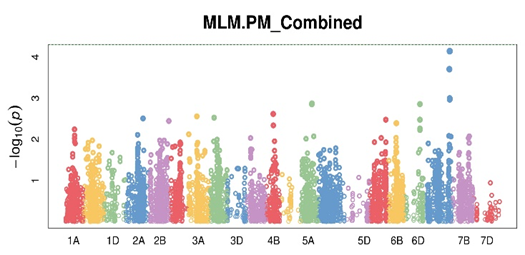

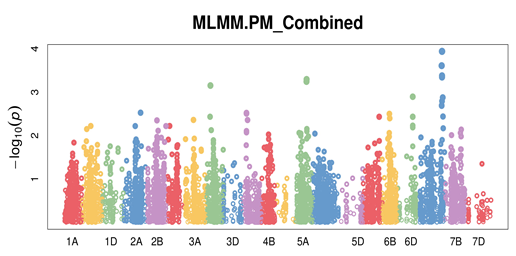

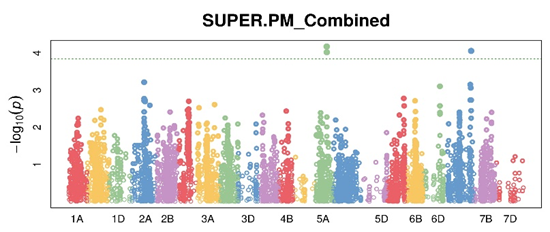

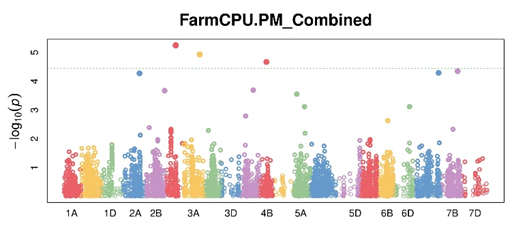

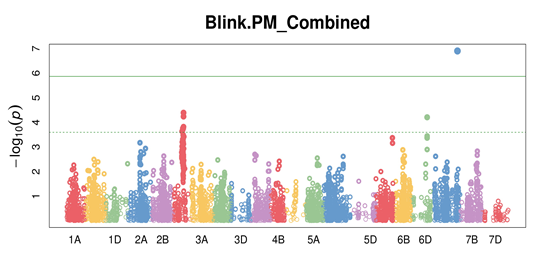


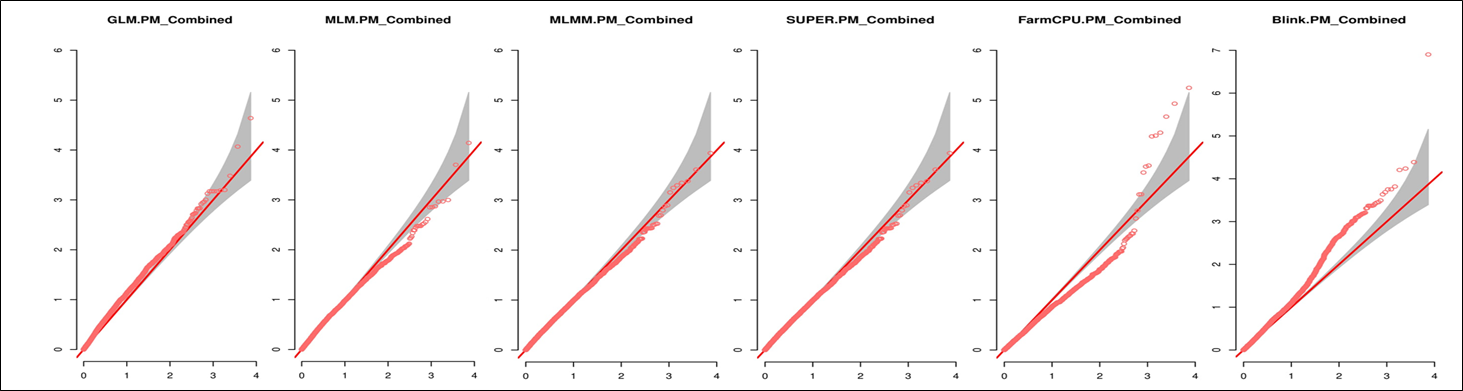


**Supplementary figure 5F**. Manhattan and Q-Q plots of the GWAS result for STB disease score combined from five environments tested at four locations. The horizontal dash and solid lines indicate the FDR adjusted P-value at 0.01 and Bonforreni corrected P-value at 0.01, respectively.
