# Supplementary material for: Genome-Wide Association Analysis and Genomic Prediction for Adult-Plant Resistance to Septoria Tritici Blotch and Powdery Mildew in Winter Wheat
Source: Front Genet. 2021 May 12;12:661742. doi: 10.3389/fgene.2021.661742 (PMC8149967; doi:10.3389/fgene.2021.661742)
Supplement: Supplementary file 1 [file Data_Sheet_1.ZIP › Supplementary files/Supplementary Table 19.docx]

Supplementary Table 19. Significant markers fitted as fixed effects in the wRR-BLUP model for genomic prediction.

| **Trait** | **Env** | **SNPs** | **Chr** | **Position** | **-LOG10(P)** |
| --- | --- | --- | --- | --- | --- |
| **STB** | **ES1** | BS00065313_51 | 5A | 63 | 4.73 |
|  |  | wsnp_Ex_c33012_41567026 | 4A | 153 | 3.71 |
|  |  | RAC875_c14309_317 | 6B | 76 | 3.68 |
|  |  | Kukri_c57014_168 | 2B | 119 | 3.53 |
|  |  | wsnp_Ex_c758_1488368 | 7B | 91 | 3.02 |
|  | **ES2** | BS00065313_51 | 5A | 63 | 4.73 |
|  |  | wsnp_CAP11_c59_99769 | 3B | 115 | 4.08 |
|  |  | Ra_c22700_818 | 5A | 83 | 3.78 |
|  |  | Ku_c24324_850 | 5A | 53 | 3.61 |
|  |  | BS00021714_51 | 1A | 78 | 3.24 |
|  | **LT2** | Ra_c69221_1167 | 5A | 42 | 4.66 |
|  |  | Excalibur_rep_c69522_83 | 1B | 171 | 4.55 |
|  |  | BS00024499_51 | 3B | 110 | 3.9 |
|  |  | wsnp_Ex_c13352_21044607 | 6B | 44 | 3.7 |
|  |  | wsnp_Ex_c16090_24522660 | 6B | 0 | 3.67 |
|  | **SW1** | Ra_c69221_1167 | 5A | 42 | 4.66 |
|  |  | wsnp_Ku_c38451_47086066 | 6A | 79 | 4.19 |
|  |  | RAC875_c23654_214 | 6B | 113 | 4.17 |
|  |  | RAC875_c75528_355 | 7A | 113 | 4.11 |
|  |  | BobWhite_c5072_107 | 2B | 67 | 3.91 |
|  |  | Kukri_rep_c101341_425 | 3B | 14 | 3.72 |
|  | **DM1** | Kukri_rep_c103893_875 | 2B | 65 | 4.11 |
|  |  | RAC875_c9770_123 | 1B | 159 | 3.4 |
|  |  | Excalibur_c15335_197 | 6B | 66 | 3.39 |
|  |  | RAC875_c27297_2153 | 2B | 99 | 3.32 |
|  |  | BS00084990_51 | 1B | 155 | 3.22 |
|  | **Combined** | wsnp_Ex_c33012_41567026 | 4A | 153 | 4.48 |
|  |  | RAC875_rep_c116515_181 | 3B | 71 | 4.06 |
|  |  | D_F5XZDLF01A85DT_301 | 1D | 61 | 3.86 |
|  |  | wsnp_Ku_c38451_47086066 | 6A | 79 | 3.73 |
|  |  | Excalibur_rep_c106935_390 | 3B | 71 | 3.7 |

Supplementary Table 19. Continued.

| **Trait** | **Env** | **SNPs** | **Chr** | **Position** | **-LOG10(P)** |
| --- | --- | --- | --- | --- | --- |
| **PM** | **ES1** | wsnp_BE445506A_Ta_2_2 | 7A | 220 | 4.79 |
|  |  | Kukri_c7605_181 | 2D | 94 | 4.10 |
|  |  | BS00077432_51 | 1A | 84 | 3.88 |
|  |  | Kukri_c11451_1882 | 7A | 228 | 3.87 |
|  |  | BS00030843_51 | 4B | 63 | 3.75 |
|  | **LT1** | wsnp_Ex_rep_c66907_65324299 | 3A | 89 | 7.33 |
|  |  | Ra_c2110_1660 | 2B | 93 | 4.91 |
|  |  | RFL_Contig2834_890 | 7A | 220 | 4.67 |
|  |  | wsnp_Ex_c6563_11378915 | 1A | 102 | 4.16 |
|  |  | RFL_Contig1338_2062 | 1D | 33 | 3.49 |
|  | **LT2** | Kukri_c6266_260 | 5A | 97 | 3.43 |
|  |  | RAC875_c31791_559 | 7B | 140 | 3.13 |
|  |  | wsnp_Ex_c790_1554988 | 5A | 98 | 3.13 |
|  |  | Excalibur_rep_c68210_468 | 6B | 72 | 3.03 |
|  |  | Kukri_rep_c70063_663 | 6A | 120 | 3.01 |
|  | **SW1** | BobWhite_c15773_166 | 2A | 144 | 15.32 |
|  |  | wsnp_Ex_c12618_20079758 | 6B | 56 | 9.71 |
|  |  | Tdurum_contig15762_139 | 6B | 70 | 7.78 |
|  |  | wsnp_Ku_rep_c73313_72887199 | 2B | 99 | 7.76 |
|  |  | Kukri_c52413_282 | 4B | 46 | 5.80 |
|  |  | Excalibur_c64302_103 | 3A | 86 | 5.35 |
|  | **DM1** | RAC875_c1357_860 | 4B | 75 | 9.5 |
|  |  | RAC875_c1742_2710 | 7B | 83 | 7.58 |
|  |  | Tdurum_contig46611_1061 | 7A | 134 | 6.7 |
|  |  | tplb0035h03_1251 | 7B | 145 | 6.62 |
|  |  | BS00101401_51 | 3A | 88 | 5.9 |
|  | **Combined** | wsnp_BE445506A_Ta_2_2 | 7A | 220 | 4.79 |
|  |  | Kukri_c7605_181 | 2D | 94 | 4.1 |
|  |  | BS00077432_51 | 1A | 84 | 3.88 |
|  |  | Kukri_c11451_1882 | 7A | 228 | 3.87 |
|  |  | BS00030843_51 | 4B | 63 | 3.75 |
